# TransFlow: a modular framework for assembling and assessing accurate de novo transcriptomes in non-model organisms

**DOI:** 10.1186/s12859-018-2384-y

**Published:** 2018-11-20

**Authors:** Pedro Seoane, Marina Espigares, Rosario Carmona, Álvaro Polonio, Julia Quintana, Enrico Cretazzo, Josefina Bota, Alejandro Pérez-García, Juan de Dios Alché, Luis Gómez, M. Gonzalo Claros

**Affiliations:** 1 Departmento de Biología Molecular y Bioquímica, Universidad de Málaga, Campus de Teatinos s/n, Malaga, 29071 Spain; 2 Plant Reproductive Biology Laboratory, Department of Biochemistry, Cell and Molecular Biology of Plants. Estación Experimental del Zaidín. CSIC, Prof. Albareda, 1, Granada, 18160 Spain; 3 Departamento de Microbiología, and Instituto de Hortofruticultura Subtropical y Mediterránea “La Mayora”, Universidad de Málaga, Consejo Superior de Investigaciones Científicas (IHSM-UMA-CSIC), Campus de Teatinos s/n, Malaga, 29071 Spain; 4 Department of Chemistry and Biochemistry, Worcester Polytechnic Institute, 100 Institute Road, Worcester, MA, 01609-2280 USA; 5 Instituto Andaluz de Investigación y Formación Agraria (IFAPA), Centro de Churriana, Cortijo de la Cruz s/n, Churriana, 29140 Spain; 6 Grup de Recerca en Biologia de les Plantes en Condicions Mediterrànies, Departament de Biologia, Universitat de les Illes Balears, Carretera de Valldemossa, km 7.5, Palma de Mallorca, 07122 Spain; 7 Departamento de Sistemas y Recursos Naturales, ETSI Forestal, de Montes y del Medio Natural, Universidad Politécnica de Madrid, Ciudad Universitaria, Madrid, 28040 Spain; 8 CBGP, INIA-Universidad Politécnica de Madrid, Campus de Montegancedo, Pozuelo de Alarcón, 28223 Spain

**Keywords:** Transcriptome, Assembling, Workflow, pipeline, PCA, Non-model organism

## Abstract

**Background:**

The advances in high-throughput sequencing technologies are allowing more and more de novo assembling of transcriptomes from many new organisms. Some degree of automation and evaluation is required to warrant reproducibility, repetitivity and the selection of the best possible transcriptome. Workflows and pipelines are becoming an absolute requirement for such a purpose, but the issue of assembling evaluation for de novo transcriptomes in organisms lacking a sequenced genome remains unsolved. An automated, reproducible and flexible framework called TransFlow to accomplish this task is described.

**Results:**

TransFlow with its five independent modules was designed to build different workflows depending on the nature of the original reads. This architecture enables different combinations of Illumina and Roche/454 sequencing data, and can be extended to other sequencing platforms. Its capabilities are illustrated with the selection of reliable plant reference transcriptomes and the assembling six transcriptomes (three case studies for grapevine leaves, olive tree pollen, and chestnut stem, and other three for haustorium, epiphytic structures and their combination for the phytopathogenic fungus *Podosphaera xanthii*). Arabidopsis and poplar transcriptomes revealed to be the best references. A common result regarding de novo assemblies is that Illumina paired-end reads of 100 nt in length assembled with OASES can provide reliable transcriptomes, while the contribution of longer reads is noticeable only when they complement a set of short, single-reads.

**Conclusions:**

TransFlow can handle up to 181 different assembling strategies. Evaluation based on principal component analyses allows its self-adaptation to different sets of reads to provide a suitable transcriptome for each combination of reads and assemblers. As a result, each case study has its own behaviour, prioritises evaluation parameters, and gives an objective and automated way for detecting the best transcriptome within a pool of them. Sequencing data type and quantity (preferably several hundred millions of 2×100 nt or longer), assemblers (OASES for Illumina, MIRA4 and EULER-SR reconciled with CAP3 for Roche/454) and strategy (preferably scaffolding with OASES, and probably merging with Roche/454 when available) arise as the most impacting factors.

**Electronic supplementary material:**

The online version of this article (10.1186/s12859-018-2384-y) contains supplementary material, which is available to authorized users.

## Background

The advances in high-throughput sequencing technologies allow the scientific community to resolve biological issues that were not accessible until now. Assembling of any genome using DNA-seq, or the quicker and cheaper approach of comprehensive transcriptomes using RNA-seq, are becoming feasible for most laboratories. In fact, transcript identification and its expression quantification is in the core of many molecular biology analyses. RNA-seq approaches are clearly replacing even the microarray technology for gene expression experiments or the variant calling approaches based on exomes [1, 2]. In fact, the power of RNA-seq is that, starting from short (> 50 bp) reads, it is able to identify and quantify already-known and new transcripts. Additionally, it is well adapted to produce tentative transcriptomes from genomically-unknown organisms (called “non-model organisms (NMOs)” in this work) using libraries from different tissues and development stages. Due to the high versatility and flexibility of RNA-seq, it has overcome the genomics field and has become a standard in the life sciences research (see [3] for a review of RNA-seq best practices from the experimental design to the transcript discovery and quantification, including differential expression). Therefore, having an ad hoc tentative transcriptome is nowadays the first step in most genomic studies regarding NMOs, that are organisms where reference genome is not available or is incomplete [3].

NMOs are usually very important from the economical or ecological point of view. For example, the European chestnut (*Castanea sativa*) is a forest tree having an important impact on producing countries due to the nutritional qualities of its fruits (chestnut) [4, 5]. Chestnut also has beneficial health effects related with the presence of bioactive compounds having antioxidant, anticarcinogenic and cardioprotective properties [6]. Another example, olive tree (*Olea europaea* L.), is one of the most important oil-producing plant species all over the world. Although the genome of the ‘Farga’ cultivar has been recently sequenced [7], it is still considered a NMO since this genome corresponds to a 1000+ year-old tree that presents many differences with other olive tree cultivars (J.D. Alché and M.G. Claros, personal communication). Many sequencing efforts have been performed regarding the transcriptome of vegetative tissues, but many questions involving olive reproductive biology, including seeds, are still open [8]. A third plant species, *Vitis vinifera*, is one of the world’s most important crop plants due to the economic value of its fruit and wine production. There is a useful draft of its genome [9], but it has been demonstrated that there is an important inter-varietal variation concerning SNPs (single nucleotide polymorphisms) and CNVs (copy-number variations) [10]. As a result, the current state of grapevine transcriptome is far from being complete [11], which suggests that grapevine transcriptomes are still required. Finally, plant disease agents are another important source of NMOs. A disease affecting many plant crops of economical significance is the powdery mildew caused by *Erysiphales* [12], obligate fungal pathogens whose hallmark is the formation of a specialised structure of parasitism, called haustorium, for the acquisition of nutrients from plant cells and the delivery of virulence factors. Recently, the genomes of five powdery mildew species were sequenced, revealing that > 70 % of their genomes are repetitive sequences, which challenges genome annotation and assembling. Cucurbits are the most severely affected group by powdery mildew, but little is known of its causing agent *Podosphaera xanthii*, even though its epiphytic transcriptome (disregarding the haustorium) was recently elucidated [13].

Experimentally defining the complete transcriptome has traditionally been a slow, costly and challenging task, including the construction of full-length cDNA libraries. Thus, even if many genomes have been sequenced, only few transcriptomes have been extensively characterised [14]. But RNA-seq has transformed this in a straightforward task. For a reliable de novo transcriptome, it is always desirable to have many libraries to sequence from different experimental conditions, development stages, tissues or organs. A large amount of reads should then be processed to assemble any tentative transcriptome. This arises a new problem, since the computational time and memory requirements increase rapidly as the number of reads increases, while computational resources are often limited in laboratories. Moreover, different transcriptomes can be obtained depending on sequencing strategies and the assembler selection, resulting in different tentative transcript sets [15]. For example Trinity [16] or OASES [17] aim to draw as much information as possible, so that they are memory-intensive. Others, such as Trans-ABySS [18] and SOAPdenovo-Trans [19] are effective provided that the computer has enough memory. There are also assemblers giving a final result very close to the true transcriptome with few assembly errors, such as CAP3 [20] or Minimus [21] that are not ready for the high-throughput sequencing. The idea that mixing assemblers in a combined strategy would provide better transcriptomes also yields several tentative transcript sets that require further evaluation. Their suitability can be easily assessed when the genome is known, but NMOs do not have any reference sequence to compare with. That is why, in some cases, evaluation is performed empirically [22, 23]; in other cases, several parameters such as accuracy, completeness, contiguity, chimerism, etc., have been implemented to assess de novo transcriptome quality [15, 24–26]. However, a true objective and comprehensive method of evaluation is still absent.

In the quest of repetitivity and reproducibility, bioinformatic analyses should be designed as workflows or pipelines that can be easily reused or recycled [27], and this is becoming a normal practice nowadays [25]. Hence, the input of several libraries from different conditions passing through several combinations of assemblers can only be reliably approached using workflow managers [28]. Consequently, the objective of the present study is to obtain an automated, reproducible and flexible framework that allows generating a workflow able to produce accurate de novo transcriptomes, especially for NMOs. The proposed framework, TransFlow, contemplates the combination of several assemblers and several kinds of reads (disregarding for their origin) into several sets of tentative transcriptomes that we called ‘assemblies’. A series of evaluation parameters are then calculated to infer which one among them resembles as much as possible the same parameters measured in well-characterised transcriptomes of model organisms. Results for transcriptomes of tree plants (olive tree, chestnut and grapevine) and one powdery mildew causing agent are presented to illustrate TransFlow capabilities.

## Methods

### Raw read sources

Plant transcriptomes were obtained from Phytozome 12.1 (https://phytozome.jgi.doe.gov/pz/portal.html). Table 1 lists RNA-seq datasets used for reference transcriptome evaluation, all of them corresponding to raw 2×100 nt reads from different HiSeq machines.

**Table 1:** Comprehensive information about Phytozome’s transcriptomes (version and number of protein coding sequences) and RNA-seq datasets (library ID and number of raw reads) downloaded to evaluate the best plant reference transcriptomes in Module 4

Source	Transcriptome version	Protein-coding transcripts	Library ID	Raw reads
*Arabidopsis thaliana* seedligs	TAIR 10	35 386	SRR4897845	78 742 616
*Populus trichocarpa* leaves	3.1	63 498	SRR1030352	65 442 430
*Vitis vinifera* leaves	12X	26 346	SRR1282039	21 171 177
*Oryza sativa* grain	7.0	52 424	SRR2072478	55 814 494
*Triticum aestivum* roots	2.2	293 053	DRR003148	7 703 831
*Zea mays* roots	5b+	88 760	SRR1282039	17 003 984

Case study 1 was performed with total RNA from leaves of *Vitis vinifera* cultivars Escursach, Shyraz, Merlot, Garnacha and Callet and sequenced by Sistemas Genómicos (Valencia, Spain) providing 397 625 017 raw 2×100 nt reads. These data are available, but protected, at BioProject 392999.

Total RNA for case study 2 was obtained from mature pollen grains of the *Olea europaea* cultivar Picual as described in [8], providing a total of 216 497 raw Roche/454 paired-end reads (BioProject PRJNA287107). These data were complemented with 40 488 002 raw 2×75 nt paired-end reads from pollen, sequenced with the NextSeq 550 at the Sequencing Unit of the University of Malaga, and available, but protected, at the BioProject 392587.

For the case study 3, *Castanea sativa* total RNA from stem tissues was extracted and sequenced at Beijing Genomics Institute in a Illumina High-Seq 2000 to obtain 90 549 382 single-end reads of 50 bp, available at BioProject PRJNA392589. Additionally, a total of 263 165 raw Roche/454 reads were downloaded from NCBI with the accession SRR954861 and incorporated in the workflow.

For *P. xanthii*, 975 070 raw Roche/454 single-end reads were used from the epiphytic structures as described in [13]. This was complemented with total RNA isolated from haustoria obtained from infected zucchini cotyledons and sequenced with the NextSeq 550 at the Sequencing Unit of the University of Malaga, yielding a total of 531 447 575 raw 2×150 nt reads. All reads are available, but protected, at BioProject 393391. For this organism, two fungal transcriptome references were downloaded from Ensembl release 31. One was *Neurospora crassa* transcriptome comprising 9 866 protein-coding transcripts; library SRR100067, with 31 301 048 raw 2×75 nt reads from purified isolate, was used for evaluation in Module 4. The other reference transcriptome was from *Candida albicans*, comprising 14 217 protein-coding transcripts. The library SRR2005826, with 7 676 629 raw 2×100 nt reads from purified isolate, was used for evaluation in Module 4.

### Workflow description

TransFlow is a framework developed with our workflow manager AutoFlow [28], based on Ruby scripting language. In this work, it has been executed on a SUSE Linux Enterprise Server 11SP2 with Slurm queue system and Infiniband FDR/QDR network (54/40 Gbps) consisting of 216 nodes with Intel E5-2670 2.6 GHz cores for a total of 3456 cores and 8.4 TB of RAM. TransFlow can be downloaded from https://github.com/seoanezonjic/TransFlow. It also requires the installation of AutoFlow and the bioinformatic tools included in TransFlow modules, such as SeqTrimNext [29] for pre-processing; the assemblers MIRA4 [30], EULER-SR [31], CAP3 [20], OASES [17], SOAPdenovo-Trans [19], RAY [32] and Minimus2 from the suite AMOS [33]; CD-HIT EST [34] to remove sequence redundancy; and FullLengtherNext (our functional and structural annotation tool already used in many de novo transcriptome assembling reports [to cite a few, [8, 13, 22, 23, 25]]) and BUSCO [35] for evaluation parameters. Statistical analyses are processed with the R package FactoMineR [36].

**Fig. 1:**
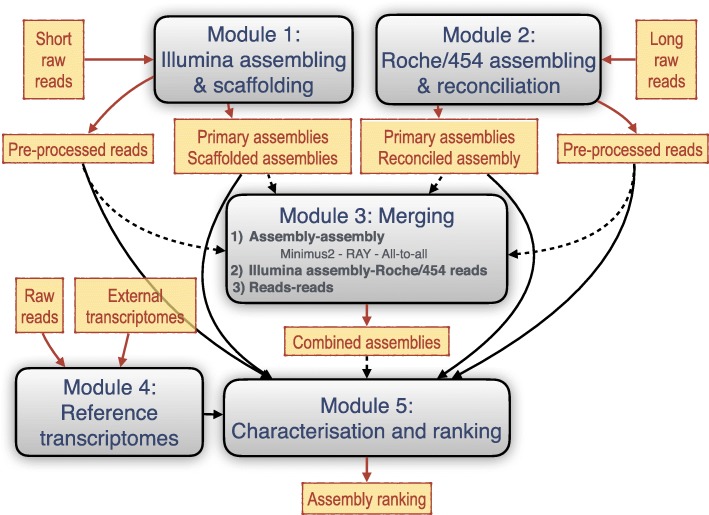
Overview and dependencies of TransFlow modules. Raw reads from sequencing platforms are used as input whether any de novo assembling is desired. Each module is independent, except for Module 5, which requires internal or external transcriptomes. Merging module (Module 3) is also optional since it is only required when combination of reads from different platforms is desired. Solid arrows, independently of their colour, indicate compulsory dependencies when the parent module is present; dashed arrows indicate optional dependencies even if the parent module is present

TransFlow comes with up to five independent modules (Fig. 1) for assembling Illumina and Roche/454 reads (Modules 1 and 2, respectively), optional combination of both technologies (Module 3), assembly characterisation and ranking (Modules 4 and 5). Modules are independent and their presence depends on the nature of the reads. A detailed description of modules is below. Estimates execution times are quite variable, since they depend on the class and number or reads, the number of modules and the number of cores used; our longer executions take 4–5 days using up to 200 (or more) cores. Concerning the disk space, it will also depend on analysed data, but in some cases up to 4 TB of disk were temporarily allocated.

#### Module 1: Illumina assembling and scaffolding

Illumina raw reads are the input for this module, where reads are pre-processed with SeqTrimNext using the Illumina built-in profile. Three different assemblers (OASES, SOAPdenovo-Trans and RAY) are then executed with *k*-mer 25 and 35 for contig assembling to obtain the corresponding “primary assemblies” (‘ct’ tag, *k*-mer and the assembler name are added to the assembly name). The subsequent scaffolding step of assemblers is then allowed (‘sc’ tag is added to the assembly name) to give the so called “scaffolded assemblies” (Fig. 1). A new non-redundant assembly is obtained using CD-HIT with a identity threshold of 100% (‘cd’ tag is added to its name). Non-redundant assemblies are reconciled using Minimus2 with default parameters to provide a set of longer contigs for each pair of *k*-mers (‘rc’ tag is added to the assembly name).

#### Module 2: Roche/454 assembling and reconciliation

Again, this module starts cleaning the raw reads using SeqTrimNext with the specific built-in profile for Roche/454 reads. Two different assemblers were used with the pre-processed reads: MIRA4 (an overlap-layout-consensus assembler) executed using RNAseq settings, and EULER-SR (a de Bruijn graph assembler) executed using a *k*-mer 29, producing “primary assemblies” (‘ct’ tag and the assembler name are added to the assembly name). The resulting contigs of both assemblers are reconciled with CAP3 to generate a “reconciled assembly” (‘rc’ tag is added to the assembly name), that is expected to improve the primary assemblies, as previously described [8, 23].

#### Module 3: merging

The aim of this module is to merge data from Roche/454 and Illumina platforms to produce a set of “combined assemblies” (Fig. 1) that should be better than each non-merged assembling itself. This is why it can only be enabled when both Module 1 and Module 2 are also enabled. Three different and simultaneous approaches are contemplated for merging: 1) assembling-assembling (aa), 2) Illumina assembling-Roche/454 reads (ar), and 3) reads-reads (rr). Hence, ‘aa’, ‘ar’ or ‘rr’ tag, respectively, is added to the assembly name.

##### Assembly-assembly.

This approach serves to merge each Illumina primary and scaffolded assembly of Module 1 with primary and reconciled assemblies of Module 2. Two combinations are produced, one using Minimus2 with default parameters, and another using RAY with *k*-mers 25 and 35. Another combined assembly is obtained after redundancy removal of all Illumina primary assemblies using CD-HIT EST, and merging with the Roche/454 reconciled assembly using Minimus2. The last combined assembly is performed as stated above, but using scaffolded assemblies instead of primary assemblies.

##### Illumina assembly-Roche/454 reads.

Every Illumina primary and scaffolded assembly of Module 1 are merged with the Roche/454 pre-processed reads of Module 2 using MIRA4 (considering the contigs as if they were Sanger sequences), as well as using RAY with *k*-mers 25 and 35. Consequently, one combined assembly is obtained for each Illumina primary and scaffolded assembly.

##### Reads-reads.

In this case, pre-processed reads from both Module 1 and Module 2 are directly assembled together using the RAY assembler with *k*-mers 25 and 35, since it is the only assembler among those described above that can successfully handle this huge amount of reads.

#### Module 4: reference transcriptomes

This module was intended to configure well-characterised reference transcriptomes from public databases for the comparative evaluation of the last module. Reference transcriptomes, besides belonging to closely related species with respect to the testing assemblies, should derive from well known genomes, although any other draft transcriptome can also be used. RNA-seq raw reads for each reference transcriptome are also required. Every reference transcriptome is analysed using Full-LengtherNext to obtain the corresponding “evaluation parameters” described in Table 2.

**Table 2:** Evaluation parameters used in TransFlow, described by their name, the software that calculates the parameter (FLN: Full-LengtherNext), a brief description of its meaning and the expected trend for such a parameter

Parameter name	Software	Description	Trend^a^
AllTransSize	FLN	The sum of every transcript length in nucleotides	*↓*
N50	FLN	The shortest contig(or scaffold) length (in nucleotides) in the set needed to cover 50% of AllTransSize	*↑*
N90	FLN	The shortest contig (or scaffold) length (in nucleotides) in the set needed to cover 90% of AllTransSize	*↑*
Contigs	FLN	Number of contigs mapping at least one pair of reads	*↓*
Contigs500	FLN	Same as previous, but taking into account only contigs > 500 nt	*↑*
MeanContigLen	FLN	Mean sequence length (in nucleotides) across all useful contigs or scaffolds	*↑*
Ns	FLN	Number of Ns (indeterminations) in the contigs or scaffolds	*↓*
MeanGapLen	FLN	Mean indetermination length in nucleotides, where 1 indicates that gaps are randomly distributed, and greater values indicate real gaps	*↓*
DiffProts	FLN	Number of unique, different proteins	*↑*
DiffComplProts	FLN	Same as previous, but onlyconsidering those proteins that seem to be complete	*↑*
MissAssembl	FLN	Percentage of contigs where the annotating protein finds similarity in both plus and minus strands	*↓*
MeanContigCov	FLN	Fraction of the contig lengths (expressed as percentage) covered by mapped reads. This fraction is calculated per contig an then averaged for the full assembly	*↑*
ComplOrtho	BUSCO	Percentage of OrthoDB orthologues from a lineage fully identified in one single contig	*↑*
FragOrtho	BUSCO	Percentage of OrthoDB orthologues from a lineage that are fragmented across several contigs	*↑*
DuplOrtho	BUSCO	Percentage of OrthoDB orthologues from a lineage that are repeated in several contigs	*↓*

All parameters are calculated for every assembly

^a^ *↑* indicates that the higher the value, the better the transcriptome; *↓* indicates that this value should be maintained in good transcriptomes as low as possible

#### Module 5: characterisation and ranking

This module is executed for every primary, scaffolded, reconciled or combined assembly loaded from any of the previous modules to provide the evaluation parameters listed in Table 2. The first set of parameters is obtained using Full-LengtherNext with full-length proteins from a specific organism division from SwissProt for assembly integrity characterisation. Note that it is not executed on reference transcriptomes as it was already done on Module 4. The last evaluation parameters of Table 2 were calculated using BUSCO for the measurement of assembly completeness by searching near-universal single-copy orthologues selected from OrthoDB [37] for a specific lineage.

For subsequent statistic analysis about factor impact on the assembling, every de novo assembly is further featured with qualitative factors referred to: 1) the ‘program’ used in the last step of assembling, 2) the ‘task’ performed, that is, the aim pursued with the program execution, 3) the ‘*k*-mer’ used in the assembling step, and 4) the ‘platform’ to reflect the way the nucleic acids were sequenced. A summary Table gathering features and evaluation parameters for every assembly is constructed, where every factor specifies the particular ‘category’ used. This table is investigated with a principal component analysis (PCA) using FactoMineR [36], an R package dedicated to multivariate data analysis. This package allows to perform a PCA automatically onto the data and can use supplementary data for individuals to facilitate the PCA interpretation. It also includes the ability of exploring similarities between individuals through hierarchical clustering on principal components (HCPC), offering the optimal number of clusters on the basis of the variance difference between clusters [38]. Every assembly on the summary Table is considered one individual, and the reference transcriptomes from Module 4 are supplementary individuals, which provide a reference for assembly evaluation. Those supplementary individuals do not affect the PCA structure since their evaluation parameters are introduced in the PCA function after being calculated for a particular set of assemblies, avoiding an increased variance due to differences between testing assemblies and reference transcriptomes. The PCA and subsequent analysis were performed keeping the first three components as they can explain until the 85% of the observed variance between individuals. One more capability of FactoMineR is the correlation analysis [38] using all coordinates of the test assemblies in the PCA space. This correlation analysis is executed between each evaluation parameter and the coordinates of the individuals for each one of the PCA components, giving the correlation coefficient (*R*) and significance (*P*) for each pair of evaluation parameter-PCA component. By default, all pairs with *P*>0.05 are discarded. Finally, the PCA results are subjected to HCPC to cluster assembling approaches. The HCPC was performed with default FactoMineR values: Euclidean distance, Ward linkage, and the optimal number of clusters computed by the HCPC function. The resulting dissimilarity matrix serves for objective assembly ranking based on averaged distances to reference transcriptomes. This matrix allows to compute the Euclidean distance from each assembly to each reference transcriptome. The Mean Distance (MD) is then calculated along the transcriptome references for each test assembly. It is assumed that the closer the assembly parameters to the references are, the better the quality of the assembly and the MD will be close to 0.

### TransFlow customisation before execution

Customisation mainly refers to modules to be executed by TransFlow, execution parameters for assemblers (and others), as well as the files with reads. For convenience, all customisable variables are included within the file called *launch_TransFlow.sh*. Commonly editable variables are: 1) *TEMPLATES* (to indicate the modules that will be used), 2) *reference* (path to folder containing the fasta file(s) for one or more reference transcriptomes), 3) *reads* (path to folder containing the fastq files that will be mapped against the reference transcriptomes, 4) *read_454* (file path to the 454/Roche reads), 5) *ill_type* (type of Illumina reads: paired or single), 6) *read_illumina_pair_1 - read_illumina_pair_2 / single_illumina*: path to Illumina paired/single files, respectively, 7) *BUSCO_DB* (specific lineage for BUSCO), 8) *FLN_DB* (database name for Full-LengtherNext), 9) *kmers*, and 10) *key_organisms* (identifiers from the assembly summary Table used as reference transcriptomes). Regarding *k*-mers, from one single value to a set of values can be indicated. An example of this file is downloaded with the TransFlow scripts (https://github.com/seoanezonjic/TransFlow/blob/master/launch_TransFlow.sh). Once customised, *launch_TransFlow.sh* is launched, and it executes AutoFlow to manage TransFlow modules.

### TransFlow output

All results are packed in an interactive HTML report that can be conveniently inspected to choose the most suitable assembly; reports for the three case studies and for *Podosphaera xanthii* transcriptomes are given is Additional files 1, 2, 3 and 4, respectively. The first image correspond to a heatmap of the evaluation parameters as a percent of the maximum value for all assemblies, including reference transcriptomes. Influencing parameters of Table 2 for each assembly can be inspected graphically, and the clusters of assemblies arises at the first sight. On the right of the heatmap, all PCA results and the HCPC grouping are embebed as a PDF document; some of these figures have been used for the main text of this document. Then, a series of tables are given, the first with the assembly ranking, another with the content of every assembly cluster, and the others correspond to evaluation parameters and factors, together with their weights, of the first two dimensions of the PCA. Finally, the values of each evaluation parameter for all assemblies are represented as interactive histograms, allowing users to obtain the original data, images, or change the default plot design.

## Results and discussion

### Plant transcriptome references

**Fig. 2:**
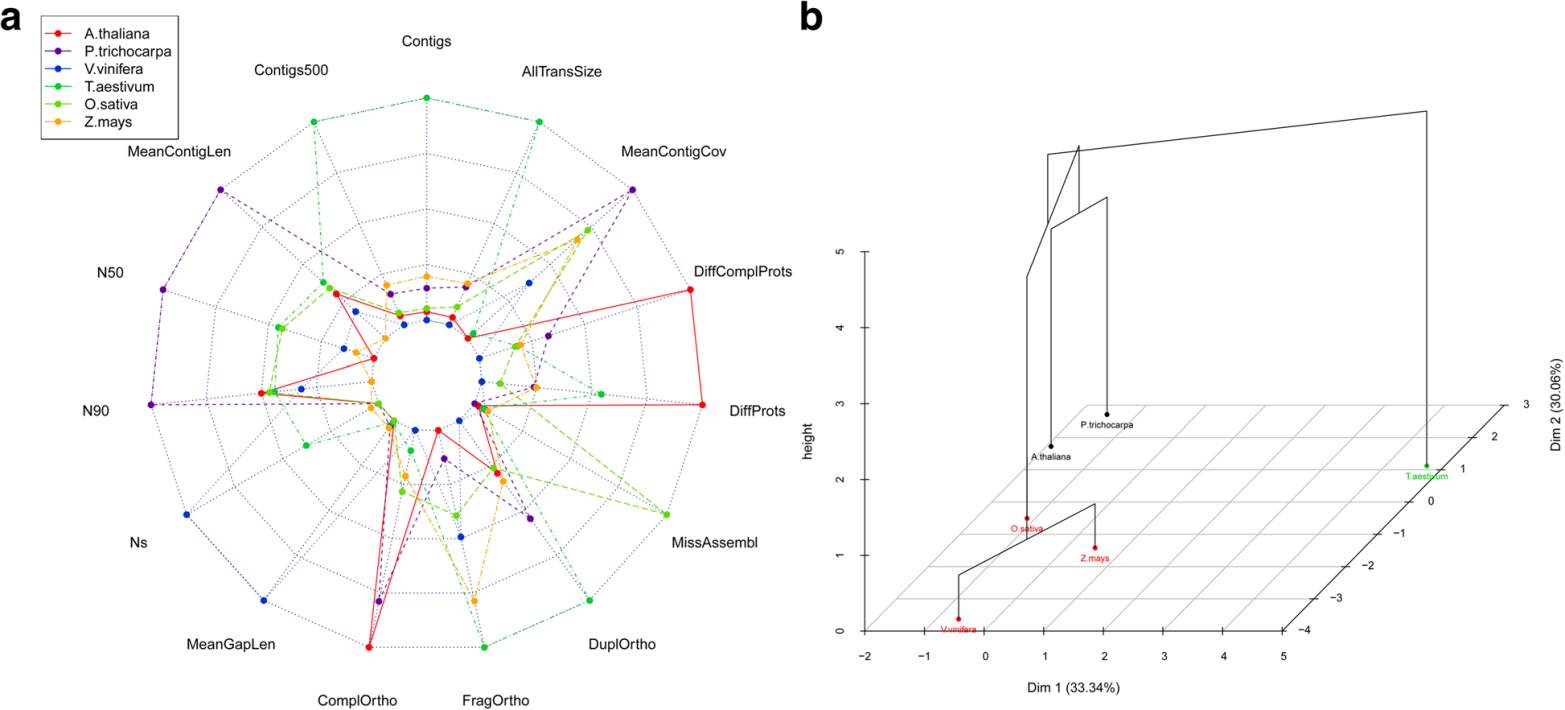
Classification of candidates to plant reference transcriptomes based on the evaluation parameters of Table 2. **a** Radar plot of evaluation parameters for the six plant transcriptomes analysed in this work: Arabidopsis (*Arabidopsis thaliana*), poplar (*Populus trichocarpa*), grapevine (*Vitis vinifera*), wheat (*Triticum aestivum*), rice (*Oryza sativa*) and maize (*Zea mays*). **b** Dendrogram on the two first dimensions of the PCA, coloured by cluster

High-quality reference transcriptomes are required for accurate ranking of assemblies. Model transcriptomes for plants were chosen among well charaterised plant species, such as Arabidopsis (*Arabidopsis thaliana*), grapevine (*Vitis vinifera*), wheat (*Triticum aestivum*), rice (*Oryza sativa*), poplar (*Populus trichocarpa*) and maize (*Zea mays*). Transcriptome sequences and raw reads were loaded into TransFlow enabling modules 4 and 5. The results shown in Fig. 2a suggest that grapevine transcriptome is not a good reference due to the high values of Ns and MeanGapLen (Table 2). Wheat transcriptome provides high values for FragOrtho, DuplOrtho, Contigs and AllTransSize, indicating that it is highly fragmented and highly redundant. The low values of N50, N90 and MeanContigLen for the maize transcriptome, combined with the high values of AllTransSize (the second highest) and FragOrtho indicate that it is a fragmented and redundant transcriptome. In the case of rice transcriptome, low values of Contigs500, DiffComplProts and ComplOrtho, together with the highest value of MissAssembl, drive to conclude that it is poor and incomplete. In contrast, Arabidopsis and poplar seem to be high-quality transcriptomes: Arabidopsis presents the highest values of ComplOrtho, DiffProts and DiffComplProts, whereas poplar transcriptome has the best values for N50, N90, MeanContigLen and MeanContigCov. In fact, the cluster-coloured PCA plot in Fig. 2b shows that both transcriptomes are grouped together and clearly distinct from the other four transcriptomes. In conclusion, transcriptomes of Arabidopsis and poplar were chosen as reference for the following case studies with plants.

### Case study 1: assembling 2×100 short paired-end reads

**Fig. 3:**
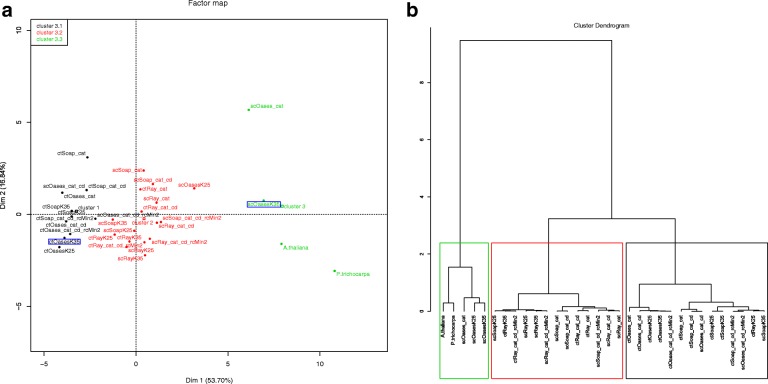
Evaluation of case study 1 (grapevine leaves) set of assemblies using PCA plots coloured by clusters. **a** PCA plot of the first two dimensions with the three significant clusters found by HCPC analysis marked as green (3.1), red (3.2) and black (3.3). The primary assembly ctOasesK35 and the scaffolded assembly scOasesK35 are boxed in blue to highlight the improvement of OASES when contigs are scaffolded. **b** Tree of assemblies where clusters are boxed in the same colours as **a**. Cluster 3.1 (green box): the 2 plant reference transcriptomes and the 3 OASES scaffolded assemblies. Cluster 3.2 (red box): 13 assemblies, 9 of them being scaffolded assemblies. Cluster 3.3 (black box): 14 assemblies, 11 of them being primary assemblies

The de novo assembling is based on one single class of reads, requiring only the addition of Module 1 to Modules 4 and 5 (as above). Grapevine leaves transcriptome was assembled from a huge amount (397 625 017) of Illumina paired-end reads. Reads were loaded into TransFlow to be first pre-processed with SeqTrimNext using a minimum read length of 95 nt. This yielded 299 905 026 pre-processed reads that, after assembling (with 250 GB of RAM required), produced a total of 30 assemblies, most of them clearly apart from those of Arabidopsis and poplar (Fig. 3; the complete report is in Additional file 1). Only scaffolded assemblies using OASES are really close to the reference transcriptomes, being the scOasesK35 assembly the closest one. In general, most scaffolded assemblies (tagged with ‘sc’) seem to be improved with respect to primary assemblies (tagged with ‘ct’), but this increase of quality is much more remarkable concerning OASES, for example when scOasesK35 position is compared to ctOasesK35 (boxed in blue in Fig. 3a). In conclusion, OASES seems to provide the best performance of all assemblers used in Module 1 (Fig. 3b and Table 3, grapevine rows), while RAY offers the poorest results.

**Table 3:** Top-five assemblies for each case study shown in this work with their respective mean distances from references (MD)

Assembly ID	Module 1	Module 2	Module 3	MD
Case study 1: Grapevine				
scOasesK35				
	OASES-scaffolded assembly with *k*-mer 35			0.383
scOasesK25				
	OASES-scaffolded assembly with *k*-mer 25			0.890
scSoap_cat_cd_rcMin2				
	Concatenation of the two SOAP-scaffolded assemblies with different *k*mers, sequence redundancy removal and Minimus2 reconciliation			1.179
scOases_cat				
	Concatenation of the two SOAP-scaffolded assemblies with different *k*mers			1.249
scRay_cat_cd_rcMin2				
	Concatenation of the two RAY-scaffolded assemblies with different *k*mers, sequence redundancy removal and Minimus2 reconciliation			1.311
Case study 2: Olive tree				
scOases_cat_cd				
	Concatenation of the two OASES-scaffolded assemblies with different *k*mers and sequence redundancy removal			0.297
aaMin2/scALL/454Cap3				
	All scafolded assemblies	CAP3-reconciled assembly	Minimus2-combined assembly of assemblies	0.323
scOases_cat				
	Concatenation of the two SOAP-scaffolded assemblies with different *k*mers			0.324
arMIRA/scOases_cat_cd				
	Concatenation of the two OASES-scaffolded assemblies with different *k*mers and sequence redundancy removal	Pre-processed reads	Assembly-reads combination using MIRA4	0.352
aaMin2/scOases_cat_cd_rcMin2/454Cap3				
	Concatenation of the two OASES-scaffolded assemblies with different *k*mers, sequence redundancy removal and Minimus2 reconciliation	CAP3-reconciled assembly	Minimus2-combined assembly of assemblies	0.367
Case study 3: Chestnut				
arMIRA/scOases_cat_cd_rcMin2				
	Concatenation of the two OASES-scaffolded assemblies with different *k*mers, sequence redundancy removal and Minimus2 reconciliation	Pre-processed reads	Assembly-reads combination using MIRA4	0.189
arMIRA/scOases_cat_cd				
	Concatenation of the two OASES-scaffolded assemblies with different *k*mers and sequence redundancy removal	Pre-processed reads	Assembly-reads combination using MIRA4	0.205
arMIRA/scOasesK25				
	OASES-scaffolded assembly with *k*-mer 25	Pre-processed reads	Assembly-reads combination using MIRA4	0.225
aaMin2/scOases_cat_cd_rcMin2/454Cap3				
	Concatenation of the two OASES-scaffolded assemblies with different *k*mers, sequence redundancy removal and Minimus2 reconciliation	CAP3-reconciled assembly	Minimus2-combined assembly of assemblies	0.269
aaMin2/scALL/454Cap3				
	All scafolded assemblies	CAP3-reconciled assembly	Minimus2-combined assembly of assemblies	0.270

### Case study 2: merging 2×75 short paired-end reads and long single reads

**Fig. 4:**
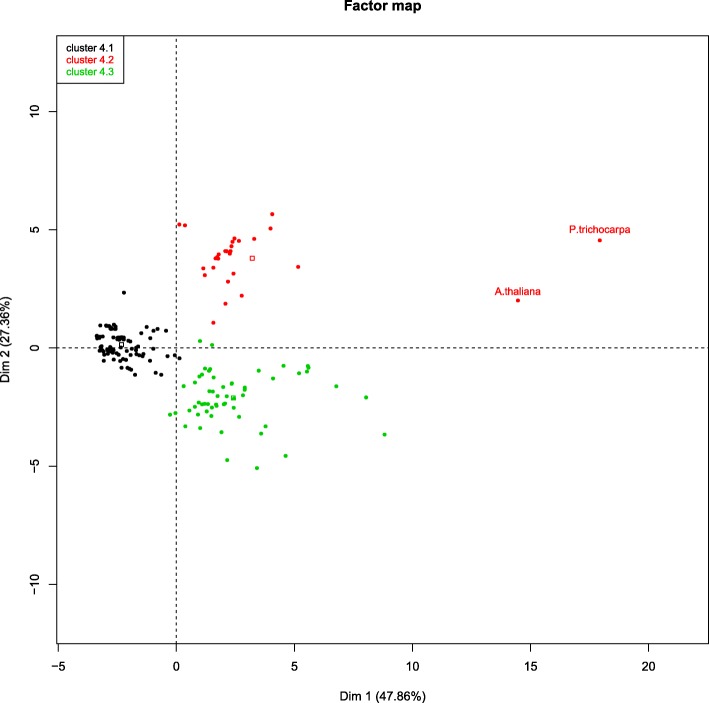
Evaluation of case study 2 (olive tree pollen) set of assemblies. PCA plot of the first two dimensions with the three significant clusters found by HCPC analysis is shown. Cluster 4.1 (red dots): the 2 plant reference transcriptomes, all Illumina assemblies combined with Roche/454 reads using MIRA4, and 2 Roche/454 assemblies (one using only MIRA4 and the other is the CAP3 reconciled assembly). Cluster 4.2 (green dots): all (primary and scaffolded) Illumina assemblies, except using RAY, executed with *k*-mer 35, the 2 Roche/454 and Illumina reads-reads merging using RAY with *k*-mers 25 and 35, all the assembly-assembly combinations using Minimus2 and the 2 all-to-all reconciliations using Minimus2. Cluster 4.3 (black dots): EULER-SR primary assembly, Illumina RAY assembly with *k*-mer 35, all the Illumina assemblies merged with Roche/454 reads using RAY, and all the assembly-assembly combinations performed with RAY

In this case, the transcriptome will be constructed using reads from two different technologies (Illumina short reads and Roche/454 long reads) from pollen of olive tree cultivar ‘Picual’. This requires the execution of TransFlow with its five modules enabled, and about 150 GB of RAM were required. The de novo assembly started with 40 488 002 Illumina paired-end and 216 497 Roche/454 single raw reads. Pre-processing of Illumina reads setting a minimum length of 65 nt provided 32 529 229 useful paired-end reads. Pre-processing of Roche/454 with default settings provided 111 242 reads. As a result of Modules 1, 2 and 3, 181 different assemblies were generated. These assemblies were grouped into three clusters (Fig. 4; the complete report is in Additional file 2). Cluster 4.1 contains the transcriptome references, scaffolded assemblies of Illumina reads merged with Roche/454 reads, and only reconciled assemblies of Roche/454 reads. This suggests that Roche/454 reads play a role in the assembling, but are not the main players. As in the case study 1, most assembling strategies including RAY appeared in the farthest cluster 4.3 (Fig. 4), indicating again that this assembler does not yield appropriate transcriptomes.

Based on the mean distances to reference transcriptomes, the top-five assemblies (Table 3, olive tree rows) present minor differences, ranging from 0.298 to 0.368. Four of them include scaffolded assemblies using OASES, being the assembly with the lowest MD that corresponds only to Illumina reads, while the others contain a combination of both Illumina and Roche/454 reads with different approaches. Interestingly, the best assembly comes from the same approach than in case study 1, while the first assembly only containing Roche/454 reads appeared at position 64 with MD = 0.708 (MIRA4 and EULER-SR primary assemblies merged with CAP3; see Additional file 2). Therefore, Illumina reads were enough to provide the best assembling, while Roche/454 reads seems to be complementary. Since these Roche/454 were the only reads used for the first pollen transcriptome [8], it is expected that the current version of the olive tree pollen has improved it. On the other hand, combining all reads with RAY does not seem again to provide successful results, since they are mainly placed in cluster 4.3.

### Case study 3: merging single, very short (50 bp) and long (Roche/454) reads

**Fig. 5:**
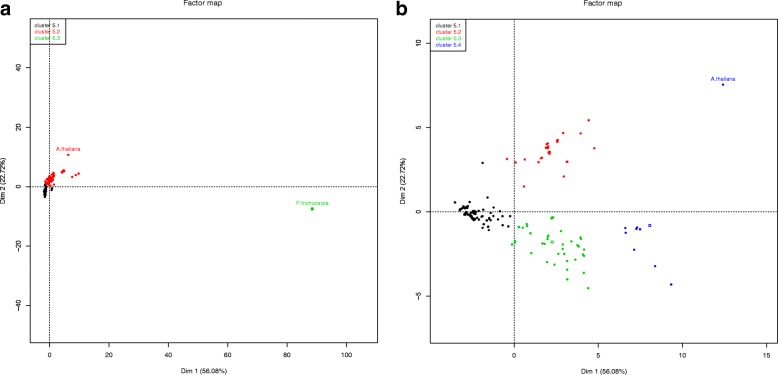
Evaluation of case study 3 (chestnut stem) set of assemblies. **a** PCA plot of the first two dimensions coloured by clusters using the two plant reference transcriptomes. Only three clusters are observed, marked as green (containing only the poplar reference transcriptome), red (containing the Arabidopsis reference transcriptome and most Illumina and Roche/454 combinations), and black. **b** PCA plot of the same data as in **a**, but using only the Arabidopsis reference transcriptome in the plot and for clustering. Four significant clusters can be now distinguished. Cluster 5.1 (blue dots) contains the Arabidopsis reference transcriptome, three assembly-assembly combinations of OASES scaffolding that includes the *k*-mer 25 with the Roche/454 reconciled assembly using Minimus2, the Minimus 2 merging of all Illumina scaffolded assemblies with the Roche/454 reconciled assembly, and four Illumina OASES-scaffolded assemblies with *k*-mer 25. Cluster 5.2 (red dots): two (MIRA4 and reconciled) Roche/454 assemblies, the all Illumina assemblies combined with Roche/454 reads using MIRA4, and the RAY assemblies with *k*-mer 35 merged with the Roche/454 reconciled assembly using Minimus2. Cluster 5.3 (green dots): all assembly-assembly combinations performed with Minimus2, except for the RAY merging using *k*-mer 25, the reads-reads combined assembly using RAY with *k*-mer 35, and the all Illumina assemblies, excepting RAY assemblies with *k*-mer 35. Cluster 5.4 (black dots): all RAY assembly-assembly combinations, Roche/454 primary assembly with EULER-SR, Illumina assemblies using RAY with *k*-mer 35 and reads-reads combination assembly using RAY with *k*-mer 35

Another interesting case to study is the de novo assembling of a transcriptome using Roche/454 long reads merged with very short and single-end Illumina reads. Again, the five modules of TransFlow are necessary, but different parametrisation for Illumina assemblers is required to deal with these single, short reads. The RAM requirements were similar to case study 2 (about 150 GB). A total of 263 165 raw Roche/454 single-end reads and 90 549 382 raw Illumina single-end reads of chestnut stem were pre-processed with default parameters, providing 147 705 Roche/454 and 88 377 297 Illumina useful reads. As in the case study 2, a total of 181 different assemblies were generated and analysed. Although poplar reference is always more distant from testing assemblies in Figs. 3 and 4 (probably due to the high number of transcripts compared to Arabidopsis), the distance to chestnut assemblies in Fig. 5a is dramatically higher and distorts statistical distances. For this reason, only Arabidopsis reference transcriptome was used for ranking in this case (Fig. 5b; the complete report is in Additional file 3), providing comparable results to case studies 1 and 2.

Four clusters can be observed in Fig. 5b. The content of cluster 5.1 suggests that Roche/454 reads are more informative for chestnut assemblies than were for olive tree (case study 2), probably due to the shorter read length and the absence of paired-reads. Moreover, most Illumina assemblies are relegated to cluster 5.3, while they appeared within cluster 4.2 in case study 2, reinforcing the idea that Illumina reads are less informative in this case. A detailed inspection of the top-five assemblies based on their distance to references (Table 3, chestnut rows) clearly shows that all of them, with MDs ranging from 0.180 to 0.230, correspond to combinations of Illumina assemblies with Roche/454 reads or assemblies. However, Roche/454 reads alone are relegated to the 44th position with MD = 0.401 (see Additional file 3), which contrasts with the 64th position with a MD = 0.708 in the case of olive tree (see above). Taken together, it can be concluded that Roche/454 reads contribution in chestnut is clearly more significant than in the case of olive tree transcriptome. As expected, 2×75 nt reads reconstructed better transcriptomes than single-end 50 nt reads, which seems to be the threshold for the requirement of longer reads (such as Roche/454) for improved transcriptomes. Finally, it also demonstrates that different raw data may require different assembling approaches.

### Fungal transcriptomes selected on biological structures

**Fig. 6:**
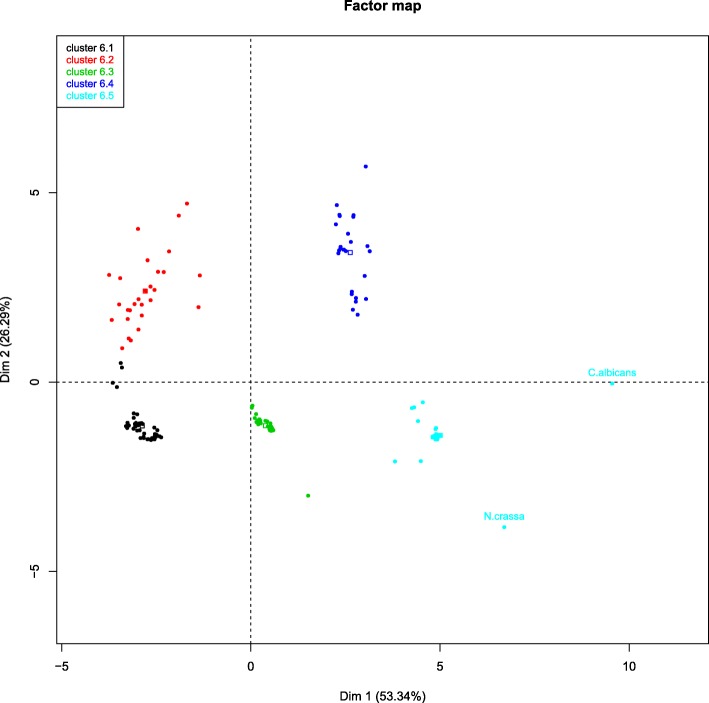
Evaluation of *P. xanthii* set of assemblies. PCA plot of the first two dimensions with the five significant clusters found by HCPC analysis is shown. Cluster 6.1 (light blue dots): fungus references, two (MIRA4-primary and reconciled) Roche/454 assemblies and all the Illumina assemblies combined with Roche/454 reads using MIRA4. Cluster 6.2 (dark blue dots): all assembly-assembly combinations using Minimus2. Cluster 6.3 (green dots): all Illumina assemblies merged with Roche/454 reads using RAY and the Roche/454 EULER-primary assembly. Cluster 6.4 (red dots): reads-reads, RAY-combined assemblies and all the Illumina assemblies, excepting the OASES-primary assemblies. Cluster 6.5 (black dots): all assembly-assembly combinations performed with RAY and OASES-primary assemblies

The versatility of TransFlow can be illustrated with the construction of several de novo transcriptomes for the fungus *P. xanthii* with different types of reads and biological structures. The aim was to obtain an accurate transcriptome for the haustorium, another for the epiphytic structures, and a comprehensive transcriptome covering both biological structures. The change of filum also required new external reference transcriptomes based on fungal species. The model organism *Candida albicans* and *Neurospora crassa* were chosen based on public read availability and transcriptome completeness. A total of 975 070 raw Roche/454 single reads were used from the epiphytic structures [13], and 531 447 575 raw Illumina 2×150 nt reads from low quality RNA extractions from isolated hautoria (A. Polonio and A. Pérez-García, personal communication). As in case studies 2 and 3, this required the execution of TransFlow with its 5 modules. Pre-processing tasks were done with default parameters, providing 687 517 Roche/454 and 140 862 905 Illumina pre-processed reads. The fact that only a 26.5% of Illumina reads were useful confirmed the RNA purification difficulties from haustoria. The 181 assemblies obtained were closer to reference transcriptomes than in previous case studies (compare axis ranges in Fig. 6 compared to previous figures). This indicated that the quality of reconstructed transcriptomes for *P. xanthii* is appropriate.

Five clusters were defined (Fig. 6; the complete report is in Additional file 4), where cluster 6.1, containing the transcriptome references, was similar to cluster 4.1. In contrast to case studies 2 and 3, the last two clusters (6.4 and 6.5) comprise most Illumina assemblies, while Roche/454 reads are more prominent in clusters 6.1 to 6.4. Moreover, in the top-five assemblies of the complete organism (Table 4, comprehensive transcriptome rows), the combined assemblies produced from Module 3 are the nearest to the reference transcriptomes, being the only-Illumina assemblies the most distant. Taken together, these results suggest that Illumina reads in this experiment yielded a poor transcriptome, while Roche/454 reads make the main contribution. This apparently contradictory behaviour may be related to the original quality of RNA used for sequencing, or alternatively to the putative low contribution of haustorium to the comprehensive transcriptome. Finally, data presented in Table 4 also serve to decide that the best haustorium transcriptome (where only Illumina reads are available) is again a scaffolded assembly using OASES, and that the best epiphytic transcriptome (made only from Roche/454 reads) was obtained after the reconciliation of MIRA4 and EULER-SR primary assemblies using CAP3, as was empirically performed in the original study [13].

**Table 4:** Top-five assemblies by biological structure for *P. xanthii* with their respective mean distances from references

Assembly ID	Module 1	Module 2	Module 3	MD
Haustorium				
scOases_cat_cd_rcMin2				
	Concatenation of the two OASES-scaffolded assemblies with different *k*mers, sequence redundancy removal and Minimus2 reconciliation			0.3848
scOases_cat				
	Concatenation of the two OASES-scaffolded assemblies with different *k*mers			0.3850
scOases_cat_cd				
	Concatenation of the two OASES-scaffolded assemblies with different *k*mers and sequence redundancy removal			0.3861
scSoap_cat_cd				
	Concatenation of the two SOAP-scaffolded assemblies with different *k*mers and sequence redundancy removal			0.3887
scOasesK35				
	OASES-scaffolded assembly with *k*-mer 35			0.3915
Epiphytic structures				
ctMIRA_ctEulK29_rcCAP3				
		CAP3-reconciled assembly		0.0558
ctMIRA				
		MIRA4-primary assembly		0.0688
ctEulK29				
		EULER-SR primary assembly with *k*-mer 29		0.1435
Comprehensive transcriptome				
arMIRA/scSoap_cat_cd_rcMin2				
	Concatenation of the two SOAP-scaffolded assemblies with different *k*mers, sequence redundancy removal and Minimus2 reconciliation	Pre-processed reads	Assembly-reads combination using MIRA4	0.0470
arMIRA/ctSoapK25				
	SOAP-primary assembly with *k*-mer 25	Pre-processed reads	Assembly-reads combination using MIRA4	0.0473
arMIRA/scRay_cat_cd_rcMin2				
	Concatenation of the two RAY-scaffolded assemblies with different *k*mers, sequence redundancy removal and Minimus2 reconciliation	Pre-processed reads	Assembly-reads combination using MIRA4	0.0476
arMIRA/ctRayK35				
	RAY-primary assembly with *k*-mer 35	Pre-processed reads	Assembly-reads combination using MIRA4	0.0479
arMIRA/ctSoap_cat_cd_rcMin2				
	Concatenation of the two SOAP-primary assembling *k*mers, sequence redundancy removal and Minimus2 reconciliation	Pre-processed reads	Assembly-reads combination using MIRA4	0.0479

### Is there any ‘best assembly’?

**Fig. 7:**
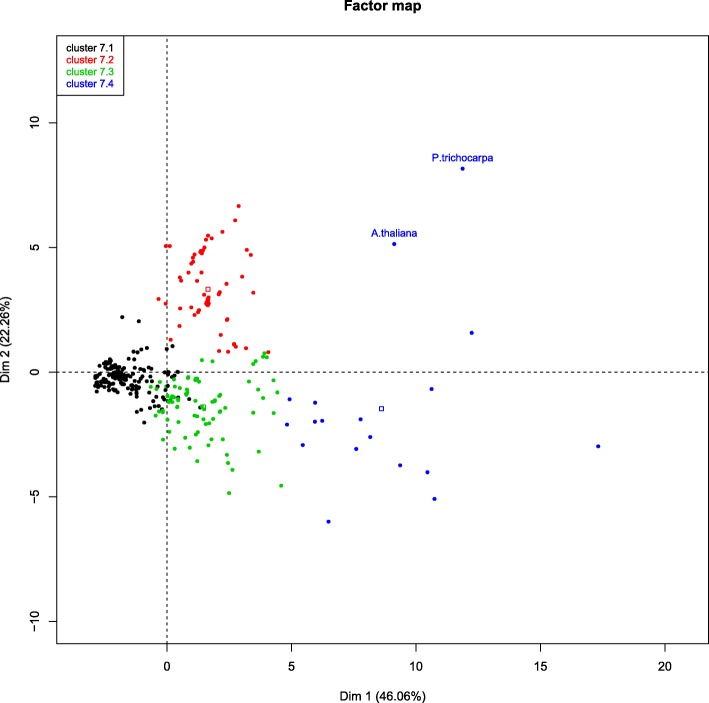
Evaluation of all plant assemblies. PCA plot of the first two dimensions with the four significant clusters found by HCPC analysis is shown. Cluster 7.1 (blue dots): plant references and most of the grapevine scaffolding assemblies. Cluster 7.2 (green dots): most of the grapevine primary assemblies, all chestnut and most of olive Minimus2 combinations, olive SOAP and OASES primary/scaffolding assemblies, most of the chestnut primary/scaffolding assemblies and the two chestnut RAY read-assembly combinations. Cluster 7.3: (red dots): all assembly-read MIRA4 combinations, all MIRA primary assemblies (and reconciliations with EULER-SR assemblies), chestnut Minimus2 combinations of RAY *k*-mer 35 primary/scaffolding assemblies, olive scaffolding assemblies that contains the *k*-mer 25 and Minimus2 combinations of them. Cluster 7.4 (black dots): all RAY assembly-assembly combinations, all EULER-SR primary assemblies, chestnut RAY primary/scaffolding assemblies, six olive Minimus2 assembly-assembly combinations and all olive Illumina primary assemblies and RAY scaffolding assemblies

All case study assemblies, that means, all plant assemblies, were analysed together and then compared to the Arabidopsis and poplar plant references (Fig. 7). Four clusters were obtained, with assemblies from the three plants distributed across all clusters. The best assembly of all is the grapevine scaffolded assembly using OASES and *k*-mer 35 (mean distance to references: 0.04); it is followed by assemblies of the other plant species (including another grapevine assembly) with distances ranging from 0.07 to 0.09 (results not shown). Even though cluster 7.1 is comprised only of grapevine assemblies, the nature of reads (long, short, paired, single...) or the number of reads to be assembled, seem to be more significant, since grapevine assemblies (the one with more Illumina “long” paired-reads) are located in the closest clusters (7.1 and 7.2), while the Roche/454 reads apear in the farthest clusters (7.3 and 7.4). The assembler is also important, since, once again, most strategies including RAY appear in the farthest cluster.

### Significant evaluation parameters

Assembling strategies based on OASES have produced suitable transcriptomes, from illumina reads alone or merged with Roche/454 long reads, depending on the nature of the original reads. Contribution of each evaluation parameter of Table 2 to the final decision was inspected based on the top-three parameters in the first two dimensions of PCA (Table 5), where FactoMineR settings give significance when *P*<0.05. Regarding plant references, the most significant parameters were only in the first dimension: Contigs, Contigs500, AllTransSize and FragOrtho, all of them being the main signs of transcriptome completeness, and absence of redundancy. This indirectly supports that the evaluation parameters were highly suitable when optimal assemblies are evaluated. Contigs500 deserves a mention, since it is also important for the first dimension when Roche/454 reads are available, and it appears in the second dimension when only Illumina reads are available. On the contrary, Contigs and AllTransSize are relegated to the second dimension, when present, for de novo assemblies. The evaluation parameters that emerge for de novo assemblies are N50 (twice in the first dimension and once in the second dimension), ComplOrth and DiffComplProts (twice in the first dimension), and N90 and MeanContigLen (once in the first dimension and twice in the second dimension). In conclusion, each case study has its own behaviour and weights differently the evaluation parameters, with a self-adapted strategy that gives an objective and automatised way for detecting suitable assemblies (transcriptomes) within a pool of them. Gene expression studies are ongoing with these suitable transcripts.

**Table 5:** Top significant assembly evaluation parameters of the first two PCA dimensions for each case study. The R coefficient measures the variable correlation with each PCA dimension

	PCA Dimension 1		PCA Dimension 2	
Case study	Name	R Coef	Name	R Coef
Plant references	Contigs	0.955	Non-significant	
	Contigs500	0.953		
	AllTransSize	0.947		
	FragOrtho	0.887		
Case study 1	N50	0.957	Contigs	0.907
(*Vitis vinifera*)	MeanContigLen	0.943	AllTransSize	0.650
	MeanContigCov	0.940	Contigs500	0.545
Case study 2	ComplOrth	0.959	N90	0.923
(*Olea europaea*)	Contigs500	0.940	MeanContigLen	0.869
	DiffComplProts	0.940	MeanContigCov	0.811
Case study 3	Contigs500	0.981	N90	0.949
(*Castanea sativa*)	ComplOrth	0.955	MeanContigLen	0.917
	DiffProts	0.937	N50	0.809
All *Podosphaera*	DiffComplProts	0.966	Contigs	0.912
*xanthii* transcriptomes	Contigs500	0.959	AllTransSize	0.801
	N50	0.936	Ns	0.780

## Conclusions

It has been shown that TransFlow can objectively assess the quality of up to 181 different assembling strategies to extract which one reconstruct a transcriptome of similar quality to an external reference. Since it is based on PCA, it is self-adapted to every set of experimental reads. It has been revealed that 2×100 nt reads (or maybe longer) and OASES assembler can provide very good transcriptomes, and that the contribution of Roche/454 reads is noticeable only when short, single-reads were used. Moreover, it seems that OASES is a good Illumina assembler and RAY is a bad transcriptome assembler. The evaluation parameters of Table 2 were accurate for reference transcriptomes, as inferred from Table 5, indicating that all assemblies analysed in this manuscript are suboptimal (assembling parameters have not been optimised for each dataset in the seek of comparison), suggesting that new assemblers or new combination strategies can improve the final transcriptome.

For convenience, most case studies have been performed with plants with the same reference transcriptome. The inclusion of a fungal study with *Neurospora crassa* and *Candida albicans* as reference transcriptomes illustrates that TransFlow depends only on the nature of reads and not the source, provided that a relatively close species can be used as transcriptome references. In fact, we are currently using TransFlow in our laboratories to assemble genomes from sole (a flatfish).

Although TransFlow has been tested here only with a limited number of assemblers, it can be customised or extended with more assemblers and more strategies. In future versions of TransFlow we plan to add a new modules capable of handle SMRT or Nanopore reads, since these technologies will become more and more present in high-throughput studies than the deprecated, although useful, Roche/454 reads. Additionally, TransFlow can be used as a benchmarking platform for assembler evaluation as follows: the combination of Modules 4 and 5, without further modification, can evaluate not only reference transcriptomes, but also several de novo transcriptomes assembled with other strategies.

The framework cannot only increase in complexity, but also in simplicity. For example, since (i) Illumina primary assemblies were always improved by scaffolded assemblies, (ii) Roche/454 primary assemblies are improved by reconciled assemblies, and (iii) approaches based on RAY are on the farthest positions, primary assemblies of Modules 1 and 2 and RAY assembler should be removed in future versions of TransFlow, while other assemblers would be included depending on literature comparisons.

The complete HTML report also offers the scientist the possibility of monitoring the evolution of assemblies, that is, if the strategy is approaching the initial assembly to the reference or not. Also, one can see if there is any factor that is clearly influencing on the strategies or the quality of reads.

## Additional files

Additional file 1 HTML report of TransFlow for Study Case 1 (grapevine). The zip file contains the elements of the report: the HTML file called *assembly_report.html* that can be open in any browser (javascript must be enabled) and inspected thoroughly; the folder *js* must be side-by-side to the HTML file for the right function. (ZIP 674 kb)

Additional file 2 HTML report of TransFlow for Study Case 2 (olive tree). The zip file contains the elements of the report: the HTML file called *assembly_report.html* that can be open in any browser (javascript must be enabled) and inspected thoroughly; the folder *js* must be side-by-side to the HTML file for the right function. (ZIP 903 kb)

Additional file 3 HTML report of TransFlow for Study Case 3 (chestnut) using only Arabidopsis as reference transcriptome. The zip file contains the elements of the report: the HTML file called *assembly_report.html* that can be open in any browser (javascript must be enabled) and inspected thoroughly; the folder *js* must be side-by-side to the HTML file for the right function. (ZIP 890 kb)

Additional file 4 HTML report of TransFlow for *P. xanthii*. The zip file contains the elements of the report: the HTML file called *assembly_report.html* that can be open in any browser (javascript must be enabled) and inspected thoroughly; the folder *js* must be side-by-side to the HTML file for the right function. (ZIP 889 kb)
