# Supplementary material for: TransFlow: a modular framework for assembling and assessing accurate de novo transcriptomes in non-model organisms
Source: BMC Bioinformatics. 2018 Nov 20;19(Suppl 14):416. doi: 10.1186/s12859-018-2384-y (PMC6245506; doi:10.1186/s12859-018-2384-y)
Supplement: Supplementary file 1 — HTML report of TransFlow for Study Case 1 (grapevine). The zip file contains the elements of the report: the HTML file called assembly_report.html that can be open in any browser (javascript must be enabled) and inspected thoroughly; the folder js must be side-by-side to the HTML file for the right function. (ZIP 674 kb) [file 12859_2018_2384_MOESM1_ESM.zip › assembly_report.html]

assembly\_report


| PCA Ranking | |
| --- | --- |
| **Name** | **PCA distance** |
| scOasesK35 | 0.3834522 |
| scOasesK25 | 0.8906528 |
| scSoap\_cat\_cd\_rcMin2 | 1.1795611 |
| scOases\_cat | 1.2495018 |
| scRay\_cat\_cd\_rcMin2 | 1.3115741 |
| scRay\_cat\_cd | 1.3138132 |
| ctRay\_cat\_cd\_rcMin2 | 1.3377686 |
| scRayK35 | 1.3533176 |
| scRay\_cat | 1.4009215 |
| ctRay\_cat\_cd | 1.4752347 |
| scSoapK25 | 1.5355467 |
| scSoap\_cat\_cd | 1.5448038 |
| ctRayK35 | 1.5695356 |
| ctRay\_cat | 1.6115329 |
| scRayK25 | 1.6864279 |
| scSoap\_cat | 1.7871883 |
| ctRayK25 | 1.8427336 |
| scSoapK35 | 1.9378203 |
| ctSoap\_cat\_cd\_rcMin2 | 2.2479149 |
| scOases\_cat\_cd\_rcMin2 | 2.2479149 |
| ctSoap\_cat\_cd | 2.5570352 |
| scOases\_cat\_cd | 2.5570352 |
| ctOases\_cat\_cd\_rcMin2 | 2.7376717 |
| ctSoapK25 | 2.7415214 |
| ctSoapK35 | 2.7530440 |
| ctSoap\_cat | 2.7914433 |
| ctOasesK35 | 2.8661377 |
| ctOases\_cat\_cd | 2.8679841 |
| ctOasesK25 | 2.9624882 |
| ctOases\_cat | 3.1016594 |

| Cluster data | | | | |
| --- | --- | --- | --- | --- |
| **Name** | **Coord Dim1** | **Coord Dim2** | **Coord Dim3** | **Cluster** |
| ctOasesK25 | -4.1270478 | 0.4530364 | -1.94664507 | 1 |
| ctOasesK35 | -3.7307088 | 0.8817145 | -1.84144893 | 1 |
| ctOases\_cat | -3.1576623 | 2.9193962 | -0.58247833 | 1 |
| ctOases\_cat\_cd | -3.3815594 | 1.5201239 | -1.31170771 | 1 |
| ctOases\_cat\_cd\_rcMin2 | -3.3563741 | 0.8486724 | -1.61659709 | 1 |
| ctSoapK25 | -2.8114332 | 0.2696603 | 0.59179299 | 1 |
| ctSoapK35 | -2.7488457 | 0.7275697 | 0.47015108 | 1 |
| ctSoap\_cat | -1.0720178 | 2.6387289 | 2.15098954 | 1 |
| ctSoap\_cat\_cd | -1.6165868 | 1.2179864 | 1.22751691 | 1 |
| ctSoap\_cat\_cd\_rcMin2 | -1.5895011 | -0.2061194 | 0.40996664 | 1 |
| scOases\_cat\_cd | -1.6165868 | 1.2179864 | 1.22751691 | 1 |
| scOases\_cat\_cd\_rcMin2 | -1.5895011 | -0.2061194 | 0.40996664 | 1 |
| ctRayK25 | -0.8416496 | -1.0717651 | -0.32299600 | 2 |
| ctRayK35 | -0.2053780 | -1.3751477 | -0.90826260 | 2 |
| ctRay\_cat | 1.2247637 | 0.4353689 | 0.91143309 | 2 |
| ctRay\_cat\_cd | 0.9303479 | -0.4686888 | 0.16711695 | 2 |
| ctRay\_cat\_cd\_rcMin2 | 0.5856051 | -1.6775641 | -0.95262832 | 2 |
| scOasesK25 | 3.8968972 | 0.8802267 | -0.95895612 | 2 |
| scRayK25 | -0.2991259 | -2.4782172 | 0.14629718 | 2 |
| scRayK35 | 0.4776387 | -2.6820935 | -0.77368883 | 2 |
| scRay\_cat | 1.9911981 | -1.2030492 | 1.50023153 | 2 |
| scRay\_cat\_cd | 1.6977029 | -2.0622722 | 0.89059760 | 2 |
| scRay\_cat\_cd\_rcMin2 | 1.0070416 | -2.1776033 | -0.15040160 | 2 |
| scSoapK25 | 0.2756926 | -1.3510016 | -0.13606863 | 2 |
| scSoapK35 | -0.6606954 | -0.7061722 | 0.53177768 | 2 |
| scSoap\_cat | 1.8157632 | 0.3485371 | 2.55451305 | 2 |
| scSoap\_cat\_cd | 2.0775128 | -0.2690156 | 1.96352977 | 2 |
| scSoap\_cat\_cd\_rcMin2 | 1.8173689 | -1.3936899 | -0.01662373 | 2 |
| A.thaliana | 7.6906617 | -3.2490395 | -2.57731207 | 3 |
| P.trichocarpa | 9.7596696 | -3.1661650 | -6.36225218 | 3 |
| scOasesK35 | 7.1206415 | 0.9026924 | -3.82281529 | 3 |
| scOases\_cat | 7.8864999 | 4.0668189 | 0.18792072 | 3 |

| PCA dimension 1 | | |
| --- | --- | --- |
| **Variables** | | |
| *Name* | *Correlation coef* | *p-valor* |
| N50 | 0.9576244 | 1.137167e-16 |
| MeanContigLen | 0.9437413 | 5.513915e-15 |
| MeanContigCov | 0.9404030 | 1.210386e-14 |
| DiffComplProts | 0.8789226 | 1.674830e-10 |
| N90 | 0.8657316 | 6.544872e-10 |
| DuplOrtho | 0.7999746 | 1.128528e-07 |
| Contigs500 | 0.7885286 | 2.278805e-07 |
| MissAssembl | 0.7426775 | 2.607987e-06 |
| AllTransSize | 0.7217300 | 6.755750e-06 |
| ComplOrtho | 0.6808016 | 3.467996e-05 |
| DiffProts | 0.6656253 | 5.969170e-05 |
| FragOrtho | -0.6554757 | 8.440639e-05 |
| **Categories** | | |
| *Name* | *Estimate* | *p-valor* |
| primary | -2.269607 | 0.0193158 |

| PCA dimension 2 | | |
| --- | --- | --- |
| **Variables** | | |
| *Name* | *Correlation coef* | *p-valor* |
| Contigs | 0.9074824 | 4.647124e-12 |
| AllTransSize | 0.6501120 | 1.008493e-04 |
| Contigs500 | 0.5458705 | 1.807021e-03 |
| FragOrtho | 0.3857312 | 3.527288e-02 |
| ComplOrtho | -0.4696318 | 8.834702e-03 |
| MeanGapLen | -0.5273004 | 2.751687e-03 |
| **Factors** | | |
| *Name* | *R2* | *p-valor* |
| Program | 0.4669321 | 0.006926167 |
| **Categories** | | |
| *Name* | *Estimate* | *p-valor* |
| cat | 1.611376 | 0.006902558 |
| join | 1.079367 | 0.014073361 |
| Ray | -1.824730 | 0.008883171 |
