# Supplementary material for: TransFlow: a modular framework for assembling and assessing accurate de novo transcriptomes in non-model organisms
Source: BMC Bioinformatics. 2018 Nov 20;19(Suppl 14):416. doi: 10.1186/s12859-018-2384-y (PMC6245506; doi:10.1186/s12859-018-2384-y)
Supplement: Supplementary file 2 — HTML report of TransFlow for Study Case 2 (olive tree). The zip file contains the elements of the report: the HTML file called assembly_report.html that can be open in any browser (javascript must be enabled) and inspected thoroughly; the folder js must be side-by-side to the HTML file for the right function. (ZIP 903 kb) [file 12859_2018_2384_MOESM2_ESM.zip › assembly_report.html]

assembly\_report


| PCA Ranking | |
| --- | --- |
| **Name** | **PCA distance** |
| scOases\_cat\_cd | 0.2975879 |
| aaMin2/scALL/454Cap3 | 0.3236560 |
| scOases\_cat | 0.3248148 |
| arMIRA/scOases\_cat\_cd | 0.3522759 |
| aaMin2/scOases\_cat\_cd\_rcMin2/454Cap3 | 0.3674497 |
| aaMin2/scOases\_cat\_cd/454Cap3 | 0.3679034 |
| scOasesK25 | 0.3853085 |
| scOases\_cat\_cd\_rcMin2 | 0.3977188 |
| arMIRA/scOasesK25 | 0.4322229 |
| aaMin2/scOasesK25/454Cap3 | 0.4325794 |
| arMIRA/scOases\_cat\_cd\_rcMin2 | 0.4344237 |
| arMIRA/scOasesK35 | 0.4753351 |
| scOasesK35 | 0.4791195 |
| aaMin2/scOasesK35/454Cap3 | 0.5067638 |
| arMIRA/scSoap\_cat\_cd | 0.5128171 |
| arMIRA/scSoap\_cat\_cd\_rcMin2 | 0.5212151 |
| arMIRA/scRay\_cat\_cd | 0.5332858 |
| arMIRA/scRay\_cat\_cd\_rcMin2 | 0.5344974 |
| arMIRA/ctRay\_cat\_cd\_rcMin2 | 0.5399172 |
| arMIRA/ctSoap\_cat\_cd\_rcMin2 | 0.5431849 |
| arMIRA/scRayK25 | 0.5442232 |
| arMIRA/ctRayK25 | 0.5459596 |
| scSoap\_cat | 0.5496921 |
| arMIRA/ctRay\_cat\_cd | 0.5516895 |
| scSoap\_cat\_cd | 0.5541983 |
| arMIRA/scSoapK35 | 0.5580152 |
| arMIRA/scSoapK25 | 0.5625724 |
| aaMin2/scSoap\_cat\_cd/454Cap3 | 0.5631574 |
| aaMin2/scSoap\_cat\_cd\_rcMin2/454Cap3 | 0.5661956 |
| arMIRA/ctSoap\_cat\_cd | 0.5666049 |
| aaMin2/ctALL/454Cap3 | 0.5815139 |
| arMIRA/scRayK35 | 0.5820523 |
| arMIRA/ctSoapK35 | 0.5847314 |
| arMIRA/ctRayK35 | 0.5868343 |
| arMIRA/ctSoapK25 | 0.5934637 |
| arMIRA/ctOases\_cat\_cd\_rcMin2 | 0.6016225 |
| aaMin2/scRay\_cat\_cd/454Cap3 | 0.6057214 |
| aaMin2/scRay\_cat\_cd\_rcMin2/454Cap3 | 0.6068391 |
| scSoap\_cat\_cd\_rcMin2 | 0.6102821 |
| arMIRA/ctOases\_cat\_cd | 0.6170148 |
| scRay\_cat | 0.6288125 |
| aaMin2/scRayK35/454Cap3 | 0.6302311 |
| ctRay\_cat | 0.6307958 |
| aaMin2/scSoapK25/454Cap3 | 0.6311146 |
| arMIRA/ctOasesK35 | 0.6311327 |
| aaMin2/scSoapK35/454Cap3 | 0.6322299 |
| arMIRA/ctOasesK25 | 0.6356415 |
| aaMin2/ctSoap\_cat\_cd\_rcMin2/454Cap3 | 0.6470715 |
| aaMin2/ctSoap\_cat\_cd/454Cap3 | 0.6519828 |
| aaMin2/scRayK25/454Cap3 | 0.6534326 |
| aaMin2/ctRay\_cat\_cd/454Cap3 | 0.6570228 |
| aaMin2/ctRay\_cat\_cd\_rcMin2/454Cap3 | 0.6625373 |
| scRay\_cat\_cd\_rcMin2 | 0.6670908 |
| aaMin2/ctRayK35/454Cap3 | 0.6691868 |
| ctSoap\_cat | 0.6733742 |
| aaMin2/ctSoapK35/454Cap3 | 0.6738998 |
| aaMin2/ctRayK25/454Cap3 | 0.6741514 |
| scRay\_cat\_cd | 0.6805568 |
| aaMin2/ctOases\_cat\_cd/454Cap3 | 0.6814427 |
| aaMin2/ctOases\_cat\_cd\_rcMin2/454Cap3 | 0.6816027 |
| ctRay\_cat\_cd | 0.6868703 |
| scSoapK25 | 0.6953073 |
| scSoapK35 | 0.7065783 |
| ctMIRA\_ctEulK29\_rcCAP3 | 0.7077450 |
| ctSoap\_cat\_cd\_rcMin2 | 0.7100766 |
| ctSoap\_cat\_cd | 0.7111838 |
| aaMin2/ctSoapK25/454Cap3 | 0.7203878 |
| ctRay\_cat\_cd\_rcMin2 | 0.7241659 |
| ctMIRA | 0.7289985 |
| rrRayK35 | 0.7370715 |
| aaMin2/ctOasesK35/454Cap3 | 0.7397927 |
| scRayK35 | 0.7512974 |
| rrRayK25 | 0.7574502 |
| ctRayK35 | 0.7582362 |
| ctOases\_cat | 0.7582455 |
| ctOases\_cat\_cd\_rcMin2 | 0.7595817 |
| aaMin2/ctOasesK25/454Cap3 | 0.7612101 |
| scRayK25 | 0.7664694 |
| ctRayK25 | 0.7707485 |
| arRayK35/scOases\_cat\_cd | 0.7759647 |
| ctOases\_cat\_cd | 0.7842556 |
| ctSoapK35 | 0.7869806 |
| arRayK35/scOasesK25 | 0.7906616 |
| aaRayK35/scOases\_cat\_cd/454Cap3 | 0.8048954 |
| aaRayK35/scOasesK25/454Cap3 | 0.8228615 |
| ctSoapK25 | 0.8238718 |
| arRayK35/scOasesK35 | 0.8386451 |
| arRayK25/scOases\_cat\_cd | 0.8466672 |
| ctOasesK35 | 0.8472273 |
| arRayK25/scOasesK25 | 0.8525290 |
| arRayK35/scOases\_cat\_cd\_rcMin2 | 0.8573477 |
| aaRayK35/scOasesK35/454Cap3 | 0.8643806 |
| aaRayK25/scOases\_cat\_cd/454Cap3 | 0.8665892 |
| ctOasesK25 | 0.8686970 |
| arRayK35/scSoap\_cat\_cd | 0.8768237 |
| aaRayK35/scOases\_cat\_cd\_rcMin2/454Cap3 | 0.8903156 |
| aaRayK25/scOasesK25/454Cap3 | 0.8963579 |
| aaRayK35/scSoap\_cat\_cd/454Cap3 | 0.9022833 |
| arRayK35/scRay\_cat\_cd | 0.9112323 |
| arRayK35/ctSoap\_cat\_cd | 0.9141015 |
| arRayK35/ctRay\_cat\_cd | 0.9203749 |
| arRayK25/scOasesK35 | 0.9227894 |
| arRayK25/scOases\_cat\_cd\_rcMin2 | 0.9248576 |
| aaRayK35/ctSoap\_cat\_cd/454Cap3 | 0.9391718 |
| aaRayK35/scRay\_cat\_cd/454Cap3 | 0.9397542 |
| ctEulK29 | 0.9420931 |
| aaRayK35/ctRay\_cat\_cd/454Cap3 | 0.9468980 |
| arRayK35/ctOases\_cat\_cd | 0.9495918 |
| arRayK25/scSoap\_cat\_cd | 0.9505967 |
| aaRayK25/scSoap\_cat\_cd/454Cap3 | 0.9612937 |
| aaRayK25/scOasesK35/454Cap3 | 0.9616382 |
| arRayK35/scRay\_cat\_cd\_rcMin2 | 0.9637355 |
| arRayK35/scSoap\_cat\_cd\_rcMin2 | 0.9657200 |
| aaRayK25/scOases\_cat\_cd\_rcMin2/454Cap3 | 0.9669999 |
| arRayK35/scSoapK35 | 0.9680253 |
| arRayK25/scRay\_cat\_cd | 0.9722662 |
| arRayK35/scSoapK25 | 0.9730568 |
| arRayK25/ctSoap\_cat\_cd | 0.9738931 |
| arRayK35/ctRayK25 | 0.9780030 |
| arRayK35/ctRay\_cat\_cd\_rcMin2 | 0.9780617 |
| arRayK35/scRayK25 | 0.9782792 |
| arRayK35/ctRayK35 | 0.9821391 |
| arRayK35/scRayK35 | 0.9829752 |
| arRayK35/ctSoapK35 | 0.9830526 |
| arRayK25/ctRay\_cat\_cd | 0.9839863 |
| aaRayK35/ctOases\_cat\_cd/454Cap3 | 0.9845061 |
| arRayK35/ctSoap\_cat\_cd\_rcMin2 | 0.9853436 |
| arRayK35/ctSoapK25 | 0.9858404 |
| arRayK35/ctOasesK35 | 0.9910695 |
| aaRayK35/scSoapK35/454Cap3 | 0.9920957 |
| arRayK35/ctOases\_cat\_cd\_rcMin2 | 0.9926237 |
| aaRayK35/scRay\_cat\_cd\_rcMin2/454Cap3 | 0.9932550 |
| aaRayK35/scSoap\_cat\_cd\_rcMin2/454Cap3 | 0.9969347 |
| aaRayK25/ctSoap\_cat\_cd/454Cap3 | 0.9979894 |
| aaRayK35/scSoapK25/454Cap3 | 0.9980091 |
| aaRayK25/scRay\_cat\_cd/454Cap3 | 0.9989472 |
| arRayK25/ctOases\_cat\_cd | 0.9990538 |
| aaRayK25/ctRay\_cat\_cd/454Cap3 | 1.0059337 |
| arRayK35/ctOasesK25 | 1.0082048 |
| aaRayK35/scRayK25/454Cap3 | 1.0105571 |
| aaRayK35/ctRayK25/454Cap3 | 1.0118493 |
| aaRayK35/ctRay\_cat\_cd\_rcMin2/454Cap3 | 1.0118553 |
| aaRayK35/scRayK35/454Cap3 | 1.0127024 |
| aaRayK35/ctSoap\_cat\_cd\_rcMin2/454Cap3 | 1.0143227 |
| aaRayK35/ctRayK35/454Cap3 | 1.0154189 |
| arRayK25/scRay\_cat\_cd\_rcMin2 | 1.0170070 |
| aaRayK35/ctSoapK35/454Cap3 | 1.0173897 |
| aaRayK35/ctSoapK25/454Cap3 | 1.0206599 |
| arRayK25/scSoapK25 | 1.0217615 |
| arRayK25/scSoap\_cat\_cd\_rcMin2 | 1.0225609 |
| arRayK25/scSoapK35 | 1.0233136 |
| aaRayK25/ctOases\_cat\_cd/454Cap3 | 1.0246271 |
| arRayK25/scRayK25 | 1.0247994 |
| arRayK25/ctRayK25 | 1.0279479 |
| arRayK25/ctRayK35 | 1.0280294 |
| arRayK25/scRayK35 | 1.0288312 |
| arRayK25/ctRay\_cat\_cd\_rcMin2 | 1.0293347 |
| arRayK25/ctSoapK25 | 1.0309612 |
| aaRayK35/ctOases\_cat\_cd\_rcMin2/454Cap3 | 1.0311420 |
| aaRayK35/ctOasesK35/454Cap3 | 1.0328926 |
| arRayK25/ctSoapK35 | 1.0348592 |
| arRayK25/ctSoap\_cat\_cd\_rcMin2 | 1.0409868 |
| arRayK25/ctOases\_cat\_cd\_rcMin2 | 1.0420995 |
| arRayK25/ctOasesK25 | 1.0434669 |
| arRayK25/ctOasesK35 | 1.0437776 |
| aaRayK25/scRayK35/454Cap3 | 1.0446008 |
| aaRayK25/ctSoapK25/454Cap3 | 1.0543207 |
| aaRayK35/ctOasesK25/454Cap3 | 1.0584347 |
| aaRayK25/ctRayK35/454Cap3 | 1.0611797 |
| aaRayK25/scRay\_cat\_cd\_rcMin2/454Cap3 | 1.0621569 |
| aaRayK25/scSoapK25/454Cap3 | 1.0655847 |
| aaRayK25/scSoapK35/454Cap3 | 1.0661011 |
| aaRayK25/ctSoapK35/454Cap3 | 1.0669443 |
| aaRayK25/scSoap\_cat\_cd\_rcMin2/454Cap3 | 1.0684275 |
| aaRayK25/ctSoap\_cat\_cd\_rcMin2/454Cap3 | 1.0703484 |
| aaRayK25/ctRayK25/454Cap3 | 1.0707742 |
| aaRayK25/scRayK25/454Cap3 | 1.0712922 |
| aaRayK25/ctRay\_cat\_cd\_rcMin2/454Cap3 | 1.0782158 |
| aaRayK25/ctOasesK25/454Cap3 | 1.0801550 |
| aaRayK25/ctOases\_cat\_cd\_rcMin2/454Cap3 | 1.0802795 |
| aaRayK25/ctOasesK35/454Cap3 | 1.0807026 |

| Cluster data | | | | |
| --- | --- | --- | --- | --- |
| **Name** | **Coord Dim1** | **Coord Dim2** | **Coord Dim3** | **Cluster** |
| aaRayK25/ctOasesK25/454Cap3 | -3.2404625 | -0.118228514 | 0.238683896 | 1 |
| aaRayK25/ctOasesK35/454Cap3 | -3.2435561 | -0.125105151 | 0.242462934 | 1 |
| aaRayK25/ctOases\_cat\_cd/454Cap3 | -2.3242948 | -0.947214932 | -0.233630946 | 1 |
| aaRayK25/ctOases\_cat\_cd\_rcMin2/454Cap3 | -3.2045480 | -0.219350089 | 0.231803865 | 1 |
| aaRayK25/ctRayK25/454Cap3 | -2.9835863 | -0.546928628 | 0.178701389 | 1 |
| aaRayK25/ctRayK35/454Cap3 | -2.7652879 | -0.830339583 | 0.026092207 | 1 |
| aaRayK25/ctRay\_cat\_cd/454Cap3 | -2.2575265 | -0.660676781 | -0.137252896 | 1 |
| aaRayK25/ctRay\_cat\_cd\_rcMin2/454Cap3 | -2.9938077 | -0.710020065 | 0.180787799 | 1 |
| aaRayK25/ctSoapK25/454Cap3 | -2.9910532 | -0.096062181 | 0.214734404 | 1 |
| aaRayK25/ctSoapK35/454Cap3 | -3.1074240 | -0.125827323 | 0.246037607 | 1 |
| aaRayK25/ctSoap\_cat\_cd/454Cap3 | -2.0427898 | -0.947666907 | -0.271599183 | 1 |
| aaRayK25/ctSoap\_cat\_cd\_rcMin2/454Cap3 | -3.1186524 | -0.186226086 | 0.236305311 | 1 |
| aaRayK25/scOasesK25/454Cap3 | -1.1374047 | -0.453847656 | -0.483884591 | 1 |
| aaRayK25/scOasesK35/454Cap3 | -1.8561423 | -0.476250669 | -0.281090773 | 1 |
| aaRayK25/scOases\_cat\_cd/454Cap3 | -0.4584707 | -1.118843668 | -0.820898821 | 1 |
| aaRayK25/scOases\_cat\_cd\_rcMin2/454Cap3 | -1.5822522 | -1.187327911 | -0.526692234 | 1 |
| aaRayK25/scRayK25/454Cap3 | -2.9865377 | -0.556816869 | 0.210563097 | 1 |
| aaRayK25/scRayK35/454Cap3 | -2.6219528 | -0.760238139 | -0.033071978 | 1 |
| aaRayK25/scRay\_cat\_cd/454Cap3 | -2.1621959 | -0.705933734 | -0.187317983 | 1 |
| aaRayK25/scRay\_cat\_cd\_rcMin2/454Cap3 | -2.8262039 | -0.713338821 | 0.086184109 | 1 |
| aaRayK25/scSoapK25/454Cap3 | -2.8831025 | -0.668191892 | 0.164726156 | 1 |
| aaRayK25/scSoapK35/454Cap3 | -2.9519054 | -0.506875496 | 0.168852192 | 1 |
| aaRayK25/scSoap\_cat\_cd/454Cap3 | -1.5088713 | -1.207404398 | -0.549451938 | 1 |
| aaRayK25/scSoap\_cat\_cd\_rcMin2/454Cap3 | -2.7307093 | -1.086220364 | 0.007053242 | 1 |
| aaRayK35/ctOasesK25/454Cap3 | -3.1849673 | 0.341322133 | 0.206319949 | 1 |
| aaRayK35/ctOasesK35/454Cap3 | -2.9433139 | 0.389367977 | 0.133420006 | 1 |
| aaRayK35/ctOases\_cat\_cd/454Cap3 | -2.1352164 | -0.382477977 | -0.286014795 | 1 |
| aaRayK35/ctOases\_cat\_cd\_rcMin2/454Cap3 | -2.9210575 | 0.374842156 | 0.137408533 | 1 |
| aaRayK35/ctRayK25/454Cap3 | -2.7020145 | 0.306190860 | 0.122551096 | 1 |
| aaRayK35/ctRayK35/454Cap3 | -2.7370611 | 0.300167643 | 0.139793968 | 1 |
| aaRayK35/ctRay\_cat\_cd/454Cap3 | -1.8807281 | -0.038339755 | -0.190527129 | 1 |
| aaRayK35/ctRay\_cat\_cd\_rcMin2/454Cap3 | -2.7135912 | 0.337230675 | 0.135831577 | 1 |
| aaRayK35/ctSoapK25/454Cap3 | -2.8274260 | 0.394356849 | 0.235368076 | 1 |
| aaRayK35/ctSoapK35/454Cap3 | -2.7874438 | 0.379964518 | 0.194350169 | 1 |
| aaRayK35/ctSoap\_cat\_cd/454Cap3 | -1.6680335 | -0.340876292 | -0.316736504 | 1 |
| aaRayK35/ctSoap\_cat\_cd\_rcMin2/454Cap3 | -2.7396186 | 0.338945798 | 0.143667748 | 1 |
| aaRayK35/scOasesK25/454Cap3 | -0.7655895 | 0.710195817 | -0.557199788 | 1 |
| aaRayK35/scOasesK35/454Cap3 | -1.2374538 | 0.692460163 | -0.425648172 | 1 |
| aaRayK35/scOases\_cat\_cd/454Cap3 | -0.1123495 | -0.326799004 | -0.771072826 | 1 |
| aaRayK35/scOases\_cat\_cd\_rcMin2/454Cap3 | -1.4049839 | 0.391759955 | -0.512489145 | 1 |
| aaRayK35/scRayK25/454Cap3 | -2.6864244 | 0.300068845 | 0.116530064 | 1 |
| aaRayK35/scRayK35/454Cap3 | -2.7049664 | 0.290165067 | 0.120445583 | 1 |
| aaRayK35/scRay\_cat\_cd/454Cap3 | -1.7948721 | -0.051821487 | -0.247841815 | 1 |
| aaRayK35/scRay\_cat\_cd\_rcMin2/454Cap3 | -2.5178798 | 0.334535621 | 0.047497886 | 1 |
| aaRayK35/scSoapK25/454Cap3 | -2.6266065 | 0.495128545 | 0.129669695 | 1 |
| aaRayK35/scSoapK35/454Cap3 | -2.5392188 | 0.429523327 | 0.051221852 | 1 |
| aaRayK35/scSoap\_cat\_cd/454Cap3 | -1.1500207 | -0.547536663 | -0.610534779 | 1 |
| aaRayK35/scSoap\_cat\_cd\_rcMin2/454Cap3 | -2.5515836 | 0.328007855 | 0.019817830 | 1 |
| arRayK25/ctOasesK25 | -2.6857088 | -0.586786987 | 0.051703205 | 1 |
| arRayK25/ctOasesK35 | -2.6771453 | -0.615160311 | 0.045044744 | 1 |
| arRayK25/ctOases\_cat\_cd | -1.9275714 | -1.213684133 | -0.375194152 | 1 |
| arRayK25/ctOases\_cat\_cd\_rcMin2 | -2.6670851 | -0.597949595 | 0.047049865 | 1 |
| arRayK25/ctRayK25 | -2.5018598 | -0.638937161 | -0.020212441 | 1 |
| arRayK25/ctRayK35 | -2.5741055 | -0.473248360 | 0.063433881 | 1 |
| arRayK25/ctRay\_cat\_cd | -1.9440129 | -0.843155783 | -0.221223186 | 1 |
| arRayK25/ctRay\_cat\_cd\_rcMin2 | -2.5446318 | -0.573579058 | 0.014937884 | 1 |
| arRayK25/ctSoapK25 | -2.5753239 | -0.543781318 | 0.050072763 | 1 |
| arRayK25/ctSoapK35 | -2.5815317 | -0.622554760 | 0.015979262 | 1 |
| arRayK25/ctSoap\_cat\_cd | -1.6778468 | -1.167737226 | -0.396144056 | 1 |
| arRayK25/ctSoap\_cat\_cd\_rcMin2 | -2.6382763 | -0.636991904 | 0.016788491 | 1 |
| arRayK25/scOasesK25 | -0.7708941 | -0.150746439 | -0.506005236 | 1 |
| arRayK25/scOasesK35 | -1.4791951 | -0.363189420 | -0.343841528 | 1 |
| arRayK25/scOases\_cat\_cd | -0.2119032 | -1.150657333 | -0.806312421 | 1 |
| arRayK25/scOases\_cat\_cd\_rcMin2 | -1.4233034 | -0.518540979 | -0.489180312 | 1 |
| arRayK25/scRayK25 | -2.4776850 | -0.619327687 | -0.016862407 | 1 |
| arRayK25/scRayK35 | -2.5865631 | -0.465133201 | 0.081514652 | 1 |
| arRayK25/scRay\_cat\_cd | -1.8115028 | -0.849926768 | -0.282924245 | 1 |
| arRayK25/scRay\_cat\_cd\_rcMin2 | -2.4211520 | -0.557974265 | -0.033600143 | 1 |
| arRayK25/scSoapK25 | -2.4466621 | -0.614991907 | -0.040410087 | 1 |
| arRayK25/scSoapK35 | -2.4738717 | -0.587234436 | -0.039887216 | 1 |
| arRayK25/scSoap\_cat\_cd | -1.3004489 | -1.383651487 | -0.631738906 | 1 |
| arRayK25/scSoap\_cat\_cd\_rcMin2 | -2.4278962 | -0.673992674 | -0.084932792 | 1 |
| arRayK35/ctOasesK25 | -2.5366924 | -0.035507508 | 0.030791986 | 1 |
| arRayK35/ctOasesK35 | -2.3611501 | -0.017720847 | -0.059236652 | 1 |
| arRayK35/ctOases\_cat\_cd | -1.6581295 | -0.606092407 | -0.450739407 | 1 |
| arRayK35/ctOases\_cat\_cd\_rcMin2 | -2.3537710 | -0.075704602 | -0.084491582 | 1 |
| arRayK35/ctRayK25 | -2.2299789 | -0.006195928 | -0.056772788 | 1 |
| arRayK35/ctRayK35 | -2.2761529 | -0.004391028 | -0.019871111 | 1 |
| arRayK35/ctRay\_cat\_cd | -1.4952873 | -0.261324671 | -0.337132807 | 1 |
| arRayK35/ctRay\_cat\_cd\_rcMin2 | -2.2420235 | 0.020574446 | -0.035751089 | 1 |
| arRayK35/ctSoapK25 | -2.3206943 | 0.006366059 | 0.012836035 | 1 |
| arRayK35/ctSoapK35 | -2.2583169 | -0.064592136 | -0.089454035 | 1 |
| arRayK35/ctSoap\_cat\_cd | -1.2964135 | -0.543986189 | -0.469651508 | 1 |
| arRayK35/ctSoap\_cat\_cd\_rcMin2 | -2.2761285 | -0.078670693 | -0.099292984 | 1 |
| arRayK35/scOasesK25 | -0.4004155 | 0.707920190 | -0.500406805 | 1 |
| arRayK35/scOasesK35 | -0.9212821 | 0.593633792 | -0.354581140 | 1 |
| arRayK35/scOases\_cat\_cd | 0.1819918 | -0.229358779 | -0.705284878 | 1 |
| arRayK35/scOases\_cat\_cd\_rcMin2 | -0.9875145 | 0.263110348 | -0.549622802 | 1 |
| arRayK35/scRayK25 | -2.2388735 | 0.006542424 | -0.038253577 | 1 |
| arRayK35/scRayK35 | -2.2884752 | 0.004395572 | -0.014487520 | 1 |
| arRayK35/scRay\_cat\_cd | -1.4275919 | -0.190091604 | -0.301910300 | 1 |
| arRayK35/scRay\_cat\_cd\_rcMin2 | -2.0992258 | 0.047871208 | -0.083136621 | 1 |
| arRayK35/scSoapK25 | -2.1978387 | 0.046379058 | -0.060082599 | 1 |
| arRayK35/scSoapK35 | -2.1320107 | 0.024511956 | -0.131604187 | 1 |
| arRayK35/scSoap\_cat\_cd | -0.8007641 | -0.669775950 | -0.698392003 | 1 |
| arRayK35/scSoap\_cat\_cd\_rcMin2 | -2.0847982 | -0.031180452 | -0.158694290 | 1 |
| ctEulK29 | -2.4982023 | 1.899528118 | 0.613999749 | 1 |
| ctRayK35 | 0.3909067 | -0.321938872 | -0.247261804 | 1 |
| A.thaliana | 13.9011302 | 4.990080588 | 0.850292011 | 2 |
| P.trichocarpa | 16.7624911 | 8.180841209 | 1.412414211 | 2 |
| arMIRA/ctOasesK25 | 0.6030097 | 3.613467252 | -0.353021527 | 2 |
| arMIRA/ctOasesK35 | 0.7267303 | 3.342841575 | -0.449948930 | 2 |
| arMIRA/ctOases\_cat\_cd | 1.4986046 | 1.452879645 | -0.727503133 | 2 |
| arMIRA/ctOases\_cat\_cd\_rcMin2 | 1.0146732 | 3.726652401 | -0.407975056 | 2 |
| arMIRA/ctRayK25 | 1.5666237 | 4.439059205 | 0.100472378 | 2 |
| arMIRA/ctRayK35 | 1.0706426 | 4.184211439 | 0.149842654 | 2 |
| arMIRA/ctRay\_cat\_cd | 1.7361994 | 3.273152909 | -0.066659610 | 2 |
| arMIRA/ctRay\_cat\_cd\_rcMin2 | 1.5715594 | 4.955547014 | 0.163120975 | 2 |
| arMIRA/ctSoapK25 | 1.0196945 | 4.123584172 | -0.148938099 | 2 |
| arMIRA/ctSoapK35 | 1.1309220 | 4.142344908 | -0.175002011 | 2 |
| arMIRA/ctSoap\_cat\_cd | 1.8263946 | 2.339733779 | -0.379892489 | 2 |
| arMIRA/ctSoap\_cat\_cd\_rcMin2 | 1.5728726 | 4.766684297 | -0.100832358 | 2 |
| arMIRA/scOasesK25 | 3.0418685 | 5.844297826 | -0.258077168 | 2 |
| arMIRA/scOasesK35 | 2.4529880 | 5.271031485 | -0.176222011 | 2 |
| arMIRA/scOases\_cat\_cd | 4.5270482 | 4.503052655 | -0.652630156 | 2 |
| arMIRA/scOases\_cat\_cd\_rcMin2 | 2.9898880 | 6.451045011 | -0.278214960 | 2 |
| arMIRA/scRayK25 | 1.5718845 | 4.551690646 | 0.110158846 | 2 |
| arMIRA/scRayK35 | 1.1091449 | 4.313350619 | 0.175652143 | 2 |
| arMIRA/scRay\_cat\_cd | 1.8910184 | 3.650561549 | 0.016455224 | 2 |
| arMIRA/scRay\_cat\_cd\_rcMin2 | 1.6238890 | 5.109182883 | 0.199450973 | 2 |
| arMIRA/scSoapK25 | 1.3598605 | 4.512994840 | -0.180105591 | 2 |
| arMIRA/scSoapK35 | 1.4163070 | 4.518719663 | -0.153096107 | 2 |
| arMIRA/scSoap\_cat\_cd | 2.4330150 | 2.814496698 | -0.424558195 | 2 |
| arMIRA/scSoap\_cat\_cd\_rcMin2 | 1.8365328 | 5.052989968 | -0.142482932 | 2 |
| ctMIRA | -0.7970216 | 5.197010725 | 0.856837547 | 2 |
| ctMIRA\_ctEulK29\_rcCAP3 | -0.5527046 | 5.212453118 | 0.730600812 | 2 |
| aaMin2/ctALL/454Cap3 | 3.3463873 | -1.298712383 | -0.402419823 | 3 |
| aaMin2/ctOasesK25/454Cap3 | 1.4663278 | -2.178398129 | -1.076580600 | 3 |
| aaMin2/ctOasesK35/454Cap3 | 1.5848476 | -1.974312286 | -0.829345685 | 3 |
| aaMin2/ctOases\_cat\_cd/454Cap3 | 2.3158763 | -1.908569741 | -0.650177072 | 3 |
| aaMin2/ctOases\_cat\_cd\_rcMin2/454Cap3 | 2.3475043 | -1.957403340 | -0.663856797 | 3 |
| aaMin2/ctRayK25/454Cap3 | 1.5318599 | -0.534135918 | 0.161284678 | 3 |
| aaMin2/ctRayK35/454Cap3 | 1.0820739 | 0.561682599 | 0.177205617 | 3 |
| aaMin2/ctRay\_cat\_cd/454Cap3 | 1.7465955 | -0.510499947 | 0.188731334 | 3 |
| aaMin2/ctRay\_cat\_cd\_rcMin2/454Cap3 | 1.7141657 | -0.591543301 | 0.199732634 | 3 |
| aaMin2/ctSoapK25/454Cap3 | 1.9815880 | -2.282932803 | -0.322606839 | 3 |
| aaMin2/ctSoapK35/454Cap3 | 2.0602732 | -1.406113414 | -0.274811098 | 3 |
| aaMin2/ctSoap\_cat\_cd/454Cap3 | 2.6241759 | -1.845171623 | -0.049787098 | 3 |
| aaMin2/ctSoap\_cat\_cd\_rcMin2/454Cap3 | 2.6638187 | -1.801552669 | -0.024969539 | 3 |
| aaMin2/scALL/454Cap3 | 7.0956718 | -0.158750100 | 1.815933755 | 3 |
| aaMin2/scOasesK25/454Cap3 | 4.7721495 | 0.275294071 | -0.394538677 | 3 |
| aaMin2/scOasesK35/454Cap3 | 3.7861316 | -0.141649960 | -0.367892159 | 3 |
| aaMin2/scOases\_cat\_cd/454Cap3 | 5.7749889 | 0.471947494 | 0.002780572 | 3 |
| aaMin2/scOases\_cat\_cd\_rcMin2/454Cap3 | 5.8120650 | 0.402374964 | 0.151867523 | 3 |
| aaMin2/scRayK25/454Cap3 | 1.9430579 | -0.845539324 | 1.213752660 | 3 |
| aaMin2/scRayK35/454Cap3 | 1.5947870 | 0.482604359 | 1.338316898 | 3 |
| aaMin2/scRay\_cat\_cd/454Cap3 | 2.7272884 | -0.947836637 | 2.313895609 | 3 |
| aaMin2/scRay\_cat\_cd\_rcMin2/454Cap3 | 2.7249542 | -0.971624833 | 2.310192604 | 3 |
| aaMin2/scSoapK25/454Cap3 | 2.6673251 | -1.494142805 | 0.326571844 | 3 |
| aaMin2/scSoapK35/454Cap3 | 2.4421031 | -1.139452487 | 0.179407691 | 3 |
| aaMin2/scSoap\_cat\_cd/454Cap3 | 3.3396050 | -0.978348698 | 0.483042106 | 3 |
| aaMin2/scSoap\_cat\_cd\_rcMin2/454Cap3 | 3.3518966 | -1.068997933 | 0.462459589 | 3 |
| ctOasesK25 | 0.5148249 | -2.712641927 | -1.565724612 | 3 |
| ctOasesK35 | 0.7275025 | -2.598498126 | -1.658486841 | 3 |
| ctOases\_cat | 3.2990851 | -4.077068224 | -2.659524380 | 3 |
| ctOases\_cat\_cd | 1.8894540 | -2.999672981 | -1.998300552 | 3 |
| ctOases\_cat\_cd\_rcMin2 | 1.6599637 | -2.477553763 | -0.937024161 | 3 |
| ctRayK25 | 0.8056412 | -1.437667813 | -0.297868515 | 3 |
| ctRay\_cat | 3.0823667 | -1.884221693 | -0.970026588 | 3 |
| ctRay\_cat\_cd | 1.9215632 | -1.418869488 | -0.547754840 | 3 |
| ctRay\_cat\_cd\_rcMin2 | 1.2363090 | -1.193531966 | 0.046115155 | 3 |
| ctSoapK25 | 1.2405495 | -3.070952796 | -1.117209187 | 3 |
| ctSoapK35 | 1.2731711 | -2.379102532 | -1.182749570 | 3 |
| ctSoap\_cat | 4.5925934 | -4.156643339 | -2.194346400 | 3 |
| ctSoap\_cat\_cd | 2.7970458 | -2.994292713 | -1.462907446 | 3 |
| ctSoap\_cat\_cd\_rcMin2 | 2.2104386 | -2.433983206 | -0.171025535 | 3 |
| rrRayK25 | 1.7272664 | -2.082937393 | 4.642897350 | 3 |
| rrRayK35 | 1.3613633 | -0.898896501 | 4.860708513 | 3 |
| scOasesK25 | 5.8118479 | 0.253985552 | -1.242368197 | 3 |
| scOasesK35 | 4.4601925 | -0.327727208 | -1.037149843 | 3 |
| scOases\_cat | 9.6015484 | -1.651265942 | -2.547171875 | 3 |
| scOases\_cat\_cd | 8.5061823 | -0.285249404 | -2.101325637 | 3 |
| scOases\_cat\_cd\_rcMin2 | 5.4759256 | 0.093324806 | -0.136594086 | 3 |
| scRayK25 | 1.6289759 | -2.125963748 | 4.624218998 | 3 |
| scRayK35 | 1.2685064 | -0.999820953 | 5.003138496 | 3 |
| scRay\_cat | 4.3205450 | -2.831532155 | 5.074265846 | 3 |
| scRay\_cat\_cd | 3.2536052 | -2.336524645 | 5.481964762 | 3 |
| scRay\_cat\_cd\_rcMin2 | 2.2483063 | -1.604081751 | 2.243759075 | 3 |
| scSoapK25 | 2.1546979 | -2.086749611 | 0.147548643 | 3 |
| scSoapK35 | 1.9701644 | -1.964643408 | -0.384797528 | 3 |
| scSoap\_cat | 5.6382226 | -3.436778579 | -0.319608663 | 3 |
| scSoap\_cat\_cd | 4.5420261 | -2.399764071 | 0.125489372 | 3 |
| scSoap\_cat\_cd\_rcMin2 | 2.9871201 | -1.528740943 | 0.452740460 | 3 |

| PCA dimension 1 | | |
| --- | --- | --- |
| **Variables** | | |
| *Name* | *Correlation coef* | *p-valor* |
| ComplOrtho | 0.9590004 | 5.939274e-100 |
| Contigs500 | 0.9407231 | 5.586685e-86 |
| DiffComplProts | 0.9402205 | 1.162692e-85 |
| DiffProts | 0.9153786 | 1.213685e-72 |
| AllTransSize | 0.9095869 | 3.486268e-70 |
| DuplOrtho | 0.8055024 | 1.552034e-42 |
| N50 | 0.6945182 | 2.170239e-27 |
| Contigs | 0.6939631 | 2.481309e-27 |
| FragOrtho | 0.5885088 | 2.982496e-18 |
| Ns | 0.5570396 | 3.839911e-16 |
| MeanContigLen | 0.4622644 | 5.718958e-11 |
| MissAssembl | 0.3390672 | 3.024597e-06 |
| MeanContigCov | 0.3177965 | 1.303915e-05 |
| N90 | 0.2922747 | 6.539620e-05 |
| MeanGapLen | 0.2027616 | 6.191304e-03 |
| **Factors** | | |
| *Name* | *R2* | *p-valor* |
| Program | 0.7740387 | 1.488776e-51 |
| Kmer | 0.5735812 | 3.222055e-28 |
| Technology | 0.3112792 | 2.795305e-14 |
| Task | 0.3599099 | 4.442467e-13 |
| **Categories** | | |
| *Name* | *Estimate* | *p-valor* |
| 25;35 | 2.6298087 | 6.263512e-14 |
| Ill | 1.4711207 | 3.100715e-13 |
| minimus | 1.3899813 | 3.158715e-12 |
| join | 2.4329041 | 4.278796e-10 |
| 29/25;35 | 2.9026036 | 3.658284e-07 |
| cat | 3.6566796 | 1.206563e-06 |
| cd-hit | 2.3859321 | 3.317503e-04 |
| mira | 0.1993934 | 9.422990e-04 |
| 454\_Ill | 3.6418408 | 5.407680e-03 |
| scaffolding | 0.8616151 | 7.183311e-03 |
| SC\_trs\_all | 5.0748894 | 7.736172e-03 |
| merge | 0.6155615 | 1.410271e-02 |
| 29/25 | 1.8973935 | 2.605421e-02 |
| oases | 1.4462113 | 2.985550e-02 |
| rs | -2.7048893 | 5.064027e-03 |
| rs\_454read/Ill | -2.7421205 | 3.084607e-03 |
| 35 | -1.7532986 | 4.187604e-06 |
| 25 | -1.9349831 | 1.046208e-07 |
| 454-Ill | -2.2511299 | 1.708075e-13 |
| Ray | -3.3897945 | 2.831653e-47 |

| PCA dimension 2 | | |
| --- | --- | --- |
| **Variables** | | |
| *Name* | *Correlation coef* | *p-valor* |
| N90 | 0.9231976 | 2.953712e-76 |
| MeanContigLen | 0.8693821 | 1.081812e-56 |
| MeanContigCov | 0.8113033 | 1.366449e-43 |
| N50 | 0.6899507 | 6.475452e-27 |
| MissAssembl | 0.6077744 | 1.163519e-19 |
| MeanGapLen | -0.1856223 | 1.235737e-02 |
| Ns | -0.2842934 | 1.050519e-04 |
| DiffProts | -0.2872305 | 8.838213e-05 |
| AllTransSize | -0.2974136 | 4.783459e-05 |
| FragOrtho | -0.5283691 | 2.098013e-14 |
| Contigs | -0.6146683 | 3.451942e-20 |
| **Factors** | | |
| *Name* | *R2* | *p-valor* |
| Program | 0.8578135 | 1.008622e-68 |
| Task | 0.3336155 | 1.074321e-11 |
| Technology | 0.2493848 | 5.102235e-11 |
| Kmer | 0.1280104 | 2.323445e-03 |
| **Categories** | | |
| *Name* | *Estimate* | *p-valor* |
| mira | 4.11439910 | 3.463688e-49 |
| rs\_454read/Ill | 1.45552394 | 4.374572e-11 |
| 454 | 3.67122154 | 3.490605e-04 |
| 29-auto | 4.11002584 | 9.704611e-03 |
| rec | 5.50948606 | 9.704611e-03 |
| cap3 | 5.08082889 | 9.704611e-03 |
| not | 4.09458345 | 9.926653e-03 |
| SOAPdenovo | -2.50698631 | 1.762075e-02 |
| rs | -0.14592366 | 1.670042e-02 |
| cd-hit | -2.20401978 | 1.065643e-02 |
| Ray | -0.47395755 | 9.614408e-03 |
| minimus | -1.16244771 | 1.395627e-03 |
| cat | -3.13787588 | 1.816584e-04 |
| join | -2.24229066 | 4.328168e-06 |
| 454-Ill | -0.09933303 | 3.992324e-06 |
| Ill | -2.41138150 | 8.918142e-10 |
