# Supplementary material for: TransFlow: a modular framework for assembling and assessing accurate de novo transcriptomes in non-model organisms
Source: BMC Bioinformatics. 2018 Nov 20;19(Suppl 14):416. doi: 10.1186/s12859-018-2384-y (PMC6245506; doi:10.1186/s12859-018-2384-y)
Supplement: Supplementary file 3 — HTML report of TransFlow for Study Case 3 (chestnut) using only Arabidopsis as reference transcriptome. The zip file contains the elements of the report: the HTML file called assembly_report.html that can be open in any browser (javascript must be enabled) and inspected thoroughly; the folder js must be side-by-side to the HTML file for the right function. (ZIP 890 kb) [file 12859_2018_2384_MOESM3_ESM.zip › assembly_report.html]

assembly\_report


| PCA Ranking | |
| --- | --- |
| **Name** | **PCA distance** |
| arMIRA/scOases\_cat\_cd\_rcMin2 | 0.1897375 |
| arMIRA/scOases\_cat\_cd | 0.2054716 |
| arMIRA/scOasesK25 | 0.2255471 |
| aaMin2/scOases\_cat\_cd\_rcMin2/454Cap3 | 0.2698252 |
| aaMin2/scALL/454Cap3 | 0.2708585 |
| aaMin2/scOases\_cat\_cd/454Cap3 | 0.2749453 |
| arMIRA/scSoap\_cat\_cd\_rcMin2 | 0.2782745 |
| arMIRA/ctSoap\_cat\_cd\_rcMin2 | 0.2785815 |
| aaMin2/scOasesK25/454Cap3 | 0.2916426 |
| arMIRA/ctSoapK25 | 0.3023859 |
| arMIRA/ctSoap\_cat\_cd | 0.3037848 |
| arMIRA/scSoapK25 | 0.3044177 |
| scOases\_cat\_cd\_rcMin2 | 0.3046110 |
| arMIRA/ctOases\_cat\_cd\_rcMin2 | 0.3062967 |
| arMIRA/scSoap\_cat\_cd | 0.3063525 |
| arMIRA/scRay\_cat\_cd\_rcMin2 | 0.3373479 |
| arMIRA/ctOases\_cat\_cd | 0.3399569 |
| arMIRA/ctRayK25 | 0.3401121 |
| arMIRA/ctRay\_cat\_cd | 0.3414097 |
| arMIRA/ctRay\_cat\_cd\_rcMin2 | 0.3425295 |
| arMIRA/scOasesK35 | 0.3435540 |
| arMIRA/ctOasesK25 | 0.3448900 |
| arMIRA/scRay\_cat\_cd | 0.3464551 |
| arMIRA/scRayK25 | 0.3501241 |
| arMIRA/ctSoapK35 | 0.3744636 |
| arMIRA/scSoapK35 | 0.3797443 |
| scOasesK25 | 0.3828282 |
| arMIRA/ctOasesK35 | 0.3934966 |
| scOases\_cat\_cd | 0.4178064 |
| aaMin2/scSoap\_cat\_cd/454Cap3 | 0.4289292 |
| aaMin2/scSoap\_cat\_cd\_rcMin2/454Cap3 | 0.4320015 |
| arMIRA/scRayK35 | 0.4370239 |
| arMIRA/ctRayK35 | 0.4370668 |
| aaMin2/scRay\_cat\_cd\_rcMin2/454Cap3 | 0.4533123 |
| aaMin2/ctRay\_cat\_cd\_rcMin2/454Cap3 | 0.4555217 |
| aaMin2/ctALL/454Cap3 | 0.4565474 |
| aaMin2/ctRayK25/454Cap3 | 0.4574757 |
| aaMin2/scRay\_cat\_cd/454Cap3 | 0.4578047 |
| aaMin2/ctRay\_cat\_cd/454Cap3 | 0.4600988 |
| aaMin2/ctOases\_cat\_cd/454Cap3 | 0.4618219 |
| aaMin2/scRayK25/454Cap3 | 0.4618479 |
| aaMin2/ctOases\_cat\_cd\_rcMin2/454Cap3 | 0.4624137 |
| aaMin2/scOasesK35/454Cap3 | 0.4645173 |
| aaMin2/scSoapK25/454Cap3 | 0.4682749 |
| aaMin2/ctSoap\_cat\_cd/454Cap3 | 0.4759070 |
| aaMin2/ctSoap\_cat\_cd\_rcMin2/454Cap3 | 0.4775170 |
| scOases\_cat | 0.4775422 |
| ctMIRA\_ctEulK29\_rcCAP3 | 0.4780426 |
| aaMin2/scRayK35/454Cap3 | 0.4851658 |
| aaMin2/ctRayK35/454Cap3 | 0.4851891 |
| scSoap\_cat\_cd\_rcMin2 | 0.5061721 |
| ctMIRA | 0.5094398 |
| aaMin2/ctSoapK25/454Cap3 | 0.5163575 |
| aaMin2/ctOasesK25/454Cap3 | 0.5189960 |
| aaMin2/scSoapK35/454Cap3 | 0.5200294 |
| aaMin2/ctSoapK35/454Cap3 | 0.5268367 |
| aaMin2/ctOasesK35/454Cap3 | 0.5343936 |
| scSoap\_cat\_cd | 0.5374805 |
| scSoap\_cat | 0.5407139 |
| ctSoap\_cat\_cd\_rcMin2 | 0.5467065 |
| ctOases\_cat\_cd\_rcMin2 | 0.5518198 |
| scOasesK35 | 0.5607122 |
| scRay\_cat\_cd\_rcMin2 | 0.5716371 |
| ctRay\_cat\_cd\_rcMin2 | 0.5716626 |
| scSoapK25 | 0.5758900 |
| rrRayK25 | 0.5765519 |
| scRay\_cat | 0.5824148 |
| ctSoap\_cat\_cd | 0.5838076 |
| ctRay\_cat | 0.5841726 |
| ctSoap\_cat | 0.5889528 |
| ctRay\_cat\_cd | 0.5997094 |
| scRay\_cat\_cd | 0.5997481 |
| ctOases\_cat\_cd | 0.6108832 |
| scRayK25 | 0.6138093 |
| arRayK35/scOases\_cat\_cd | 0.6145670 |
| ctRayK25 | 0.6164634 |
| ctEulK29 | 0.6205552 |
| ctOases\_cat | 0.6249918 |
| ctSoapK25 | 0.6294339 |
| arRayK35/scOasesK25 | 0.6432879 |
| aaRayK35/scOases\_cat\_cd/454Cap3 | 0.6455435 |
| arRayK25/scOases\_cat\_cd | 0.6486612 |
| ctOasesK25 | 0.6562250 |
| aaRayK35/scOasesK25/454Cap3 | 0.6603516 |
| rrRayK35 | 0.6642427 |
| aaRayK25/scOases\_cat\_cd/454Cap3 | 0.6681442 |
| scSoapK35 | 0.6711779 |
| arRayK25/scOasesK25 | 0.6713332 |
| ctSoapK35 | 0.6754062 |
| aaRayK25/scOasesK25/454Cap3 | 0.6817944 |
| arRayK35/scOases\_cat\_cd\_rcMin2 | 0.6873051 |
| ctOasesK35 | 0.6939338 |
| arRayK25/scOases\_cat\_cd\_rcMin2 | 0.7046351 |
| arRayK35/ctSoap\_cat\_cd | 0.7081949 |
| arRayK35/scSoap\_cat\_cd | 0.7108690 |
| aaRayK35/scOases\_cat\_cd\_rcMin2/454Cap3 | 0.7118417 |
| aaRayK25/scOases\_cat\_cd\_rcMin2/454Cap3 | 0.7232883 |
| arRayK35/ctOases\_cat\_cd | 0.7250306 |
| aaRayK35/ctSoap\_cat\_cd/454Cap3 | 0.7332836 |
| arRayK25/scSoap\_cat\_cd | 0.7340534 |
| arRayK35/scOasesK35 | 0.7347535 |
| arRayK25/ctSoap\_cat\_cd | 0.7362746 |
| aaRayK35/scSoap\_cat\_cd/454Cap3 | 0.7382777 |
| arRayK25/ctOases\_cat\_cd | 0.7425737 |
| aaRayK25/scSoap\_cat\_cd/454Cap3 | 0.7474488 |
| aaRayK25/ctSoap\_cat\_cd/454Cap3 | 0.7484871 |
| arRayK35/ctSoapK25 | 0.7513044 |
| arRayK35/scRay\_cat\_cd | 0.7514468 |
| arRayK35/ctRay\_cat\_cd | 0.7518787 |
| arRayK35/scRayK25 | 0.7549801 |
| arRayK35/ctSoap\_cat\_cd\_rcMin2 | 0.7552744 |
| arRayK35/ctRayK25 | 0.7562551 |
| scRayK35 | 0.7573912 |
| ctRayK35 | 0.7574483 |
| arRayK35/ctOases\_cat\_cd\_rcMin2 | 0.7574574 |
| arRayK35/scSoap\_cat\_cd\_rcMin2 | 0.7575884 |
| arRayK35/scSoapK25 | 0.7580582 |
| arRayK25/scOasesK35 | 0.7584233 |
| aaRayK35/ctOases\_cat\_cd/454Cap3 | 0.7587585 |
| arRayK35/ctSoapK35 | 0.7612822 |
| arRayK35/scRay\_cat\_cd\_rcMin2 | 0.7613646 |
| arRayK35/ctRay\_cat\_cd\_rcMin2 | 0.7624961 |
| aaRayK25/ctOases\_cat\_cd/454Cap3 | 0.7643659 |
| arRayK35/ctOasesK25 | 0.7657226 |
| arRayK35/scSoapK35 | 0.7666604 |
| arRayK35/ctOasesK35 | 0.7677774 |
| arRayK25/ctRay\_cat\_cd | 0.7720897 |
| arRayK25/scRay\_cat\_cd | 0.7728982 |
| arRayK25/ctSoapK25 | 0.7743402 |
| arRayK35/scRayK35 | 0.7750290 |
| aaRayK25/scOasesK35/454Cap3 | 0.7781575 |
| arRayK25/scSoapK25 | 0.7793773 |
| arRayK25/ctRayK25 | 0.7804090 |
| arRayK25/ctSoap\_cat\_cd\_rcMin2 | 0.7806754 |
| arRayK25/ctOases\_cat\_cd\_rcMin2 | 0.7812163 |
| aaRayK25/ctRay\_cat\_cd/454Cap3 | 0.7813600 |
| arRayK25/scSoap\_cat\_cd\_rcMin2 | 0.7815631 |
| arRayK25/ctSoapK35 | 0.7815807 |
| arRayK35/ctRayK35 | 0.7816987 |
| arRayK25/scRayK25 | 0.7817724 |
| aaRayK35/scOasesK35/454Cap3 | 0.7819928 |
| arRayK25/ctOasesK25 | 0.7820221 |
| aaRayK25/ctRay\_cat\_cd\_rcMin2/454Cap3 | 0.7823447 |
| aaRayK25/scRay\_cat\_cd/454Cap3 | 0.7826844 |
| aaRayK25/ctRayK25/454Cap3 | 0.7830661 |
| arRayK25/ctRay\_cat\_cd\_rcMin2 | 0.7835878 |
| arRayK25/ctOasesK35 | 0.7839193 |
| arRayK25/scRay\_cat\_cd\_rcMin2 | 0.7839432 |
| arRayK25/scSoapK35 | 0.7842604 |
| aaRayK25/scRay\_cat\_cd\_rcMin2/454Cap3 | 0.7847017 |
| aaRayK25/scSoap\_cat\_cd\_rcMin2/454Cap3 | 0.7847257 |
| aaRayK25/scRayK25/454Cap3 | 0.7874019 |
| aaRayK25/scSoapK25/454Cap3 | 0.7882649 |
| aaRayK35/scSoap\_cat\_cd\_rcMin2/454Cap3 | 0.7884148 |
| aaRayK35/ctSoap\_cat\_cd\_rcMin2/454Cap3 | 0.7892735 |
| aaRayK35/ctRay\_cat\_cd/454Cap3 | 0.7903902 |
| aaRayK35/scRay\_cat\_cd/454Cap3 | 0.7914552 |
| aaRayK35/ctOases\_cat\_cd\_rcMin2/454Cap3 | 0.7950398 |
| aaRayK35/ctSoapK25/454Cap3 | 0.7955284 |
| aaRayK35/scSoapK25/454Cap3 | 0.7956468 |
| aaRayK25/ctOases\_cat\_cd\_rcMin2/454Cap3 | 0.7956916 |
| aaRayK25/ctSoap\_cat\_cd\_rcMin2/454Cap3 | 0.7964087 |
| aaRayK35/ctRay\_cat\_cd\_rcMin2/454Cap3 | 0.7971305 |
| aaRayK25/scRayK35/454Cap3 | 0.7971424 |
| arRayK25/scRayK35 | 0.7972007 |
| aaRayK25/ctRayK35/454Cap3 | 0.7973685 |
| aaRayK25/ctSoapK25/454Cap3 | 0.7975736 |
| arRayK25/ctRayK35 | 0.7978012 |
| aaRayK35/scRay\_cat\_cd\_rcMin2/454Cap3 | 0.7978729 |
| aaRayK35/ctRayK25/454Cap3 | 0.8010640 |
| aaRayK35/scRayK25/454Cap3 | 0.8026397 |
| aaRayK25/scSoapK35/454Cap3 | 0.8032999 |
| aaRayK25/ctSoapK35/454Cap3 | 0.8062829 |
| aaRayK25/ctOasesK25/454Cap3 | 0.8095831 |
| aaRayK35/scSoapK35/454Cap3 | 0.8107360 |
| aaRayK35/ctSoapK35/454Cap3 | 0.8116658 |
| aaRayK25/ctOasesK35/454Cap3 | 0.8137893 |
| aaRayK35/ctOasesK35/454Cap3 | 0.8198544 |
| aaRayK35/ctOasesK25/454Cap3 | 0.8259497 |
| aaRayK35/scRayK35/454Cap3 | 0.8312972 |
| aaRayK35/ctRayK35/454Cap3 | 0.8316171 |

| Cluster data | | | | |
| --- | --- | --- | --- | --- |
| **Name** | **Coord Dim1** | **Coord Dim2** | **Coord Dim3** | **Cluster** |
| aaRayK25/ctOasesK25/454Cap3 | -2.8184124 | -0.4410166329 | 0.59518768 | 1 |
| aaRayK25/ctOasesK35/454Cap3 | -2.8783205 | -0.4331884883 | 0.64462289 | 1 |
| aaRayK25/ctOases\_cat\_cd/454Cap3 | -1.6998611 | -1.0834211851 | -0.42031994 | 1 |
| aaRayK25/ctOases\_cat\_cd\_rcMin2/454Cap3 | -2.6561260 | -0.4146869929 | 0.52463049 | 1 |
| aaRayK25/ctRayK25/454Cap3 | -2.4654975 | -0.4520781376 | 0.44344213 | 1 |
| aaRayK25/ctRayK35/454Cap3 | -2.7767803 | -0.2739163997 | 0.76524955 | 1 |
| aaRayK25/ctRay\_cat\_cd/454Cap3 | -2.4363617 | -0.4560480305 | 0.36414012 | 1 |
| aaRayK25/ctRay\_cat\_cd\_rcMin2/454Cap3 | -2.4102891 | -0.5172110415 | 0.39512314 | 1 |
| aaRayK25/ctSoapK25/454Cap3 | -2.7184297 | -0.3557089236 | 0.53140478 | 1 |
| aaRayK25/ctSoapK35/454Cap3 | -2.8287163 | -0.3612685901 | 0.61681221 | 1 |
| aaRayK25/ctSoap\_cat\_cd/454Cap3 | -1.6632375 | -0.8670461142 | -0.23596631 | 1 |
| aaRayK25/ctSoap\_cat\_cd\_rcMin2/454Cap3 | -2.6870580 | -0.3806749779 | 0.52046825 | 1 |
| aaRayK25/scOasesK25/454Cap3 | -1.1355426 | -0.3236276685 | -0.12964151 | 1 |
| aaRayK25/scOasesK35/454Cap3 | -2.5022954 | -0.3075126544 | 0.55901671 | 1 |
| aaRayK25/scOases\_cat\_cd/454Cap3 | -0.5280985 | -0.7993079913 | -0.52738682 | 1 |
| aaRayK25/scOases\_cat\_cd\_rcMin2/454Cap3 | -1.5117793 | -0.6229780863 | -0.04973858 | 1 |
| aaRayK25/scRayK25/454Cap3 | -2.4880633 | -0.4992085922 | 0.40142034 | 1 |
| aaRayK25/scRayK35/454Cap3 | -2.7630685 | -0.2896077808 | 0.73571639 | 1 |
| aaRayK25/scRay\_cat\_cd/454Cap3 | -2.4254448 | -0.4969857739 | 0.34845684 | 1 |
| aaRayK25/scRay\_cat\_cd\_rcMin2/454Cap3 | -2.4141506 | -0.5564073300 | 0.38588761 | 1 |
| aaRayK25/scSoapK25/454Cap3 | -2.5531618 | -0.4214161627 | 0.45344611 | 1 |
| aaRayK25/scSoapK35/454Cap3 | -2.7869075 | -0.3653713725 | 0.57923424 | 1 |
| aaRayK25/scSoap\_cat\_cd/454Cap3 | -1.6521466 | -0.8579744609 | -0.26800063 | 1 |
| aaRayK25/scSoap\_cat\_cd\_rcMin2/454Cap3 | -2.4246166 | -0.5439980900 | 0.41890679 | 1 |
| aaRayK35/ctOasesK25/454Cap3 | -3.1720535 | -0.2078486479 | 0.68796452 | 1 |
| aaRayK35/ctOasesK35/454Cap3 | -3.1377031 | -0.1421050933 | 0.70744601 | 1 |
| aaRayK35/ctOases\_cat\_cd/454Cap3 | -1.9666544 | -0.6175736106 | -0.36040297 | 1 |
| aaRayK35/ctOases\_cat\_cd\_rcMin2/454Cap3 | -2.8754955 | -0.0520749521 | 0.50044506 | 1 |
| aaRayK35/ctRayK25/454Cap3 | -2.9700848 | -0.0229417438 | 0.52996656 | 1 |
| aaRayK35/ctRayK35/454Cap3 | -3.5025859 | 0.2221760368 | 1.02682408 | 1 |
| aaRayK35/ctRay\_cat\_cd/454Cap3 | -2.8171208 | -0.0437770007 | 0.40352149 | 1 |
| aaRayK35/ctRay\_cat\_cd\_rcMin2/454Cap3 | -2.9679832 | 0.0520634625 | 0.56655884 | 1 |
| aaRayK35/ctSoapK25/454Cap3 | -2.8945764 | -0.0310664065 | 0.50213427 | 1 |
| aaRayK35/ctSoapK35/454Cap3 | -3.0598399 | -0.0992934636 | 0.63309346 | 1 |
| aaRayK35/ctSoap\_cat\_cd/454Cap3 | -1.7792956 | -0.3974319753 | -0.32605905 | 1 |
| aaRayK35/ctSoap\_cat\_cd\_rcMin2/454Cap3 | -2.8245724 | -0.0146501143 | 0.47344987 | 1 |
| aaRayK35/scOasesK25/454Cap3 | -1.1762888 | 0.1874848652 | -0.22388966 | 1 |
| aaRayK35/scOasesK35/454Cap3 | -2.7363706 | -0.0108507579 | 0.50482063 | 1 |
| aaRayK35/scOases\_cat\_cd/454Cap3 | -0.5947522 | -0.2674050311 | -0.56171164 | 1 |
| aaRayK35/scOases\_cat\_cd\_rcMin2/454Cap3 | -1.7746074 | -0.0135047000 | -0.06007056 | 1 |
| aaRayK35/scRayK25/454Cap3 | -2.9868905 | -0.0277615828 | 0.53170452 | 1 |
| aaRayK35/scRayK35/454Cap3 | -3.4957644 | 0.2171755193 | 1.01629299 | 1 |
| aaRayK35/scRay\_cat\_cd/454Cap3 | -2.8352235 | -0.0379638807 | 0.42407022 | 1 |
| aaRayK35/scRay\_cat\_cd\_rcMin2/454Cap3 | -2.9725373 | 0.0447037574 | 0.56016695 | 1 |
| aaRayK35/scSoapK25/454Cap3 | -2.8976975 | -0.0297746376 | 0.52021515 | 1 |
| aaRayK35/scSoapK35/454Cap3 | -3.0578226 | -0.0838988588 | 0.63702139 | 1 |
| aaRayK35/scSoap\_cat\_cd/454Cap3 | -1.8262868 | -0.4280611282 | -0.32290123 | 1 |
| aaRayK35/scSoap\_cat\_cd\_rcMin2/454Cap3 | -2.8229940 | -0.0004447816 | 0.48449529 | 1 |
| arRayK25/ctOasesK25 | -2.3050358 | -0.6563569649 | 0.27758402 | 1 |
| arRayK25/ctOasesK35 | -2.3499949 | -0.6286916808 | 0.29882819 | 1 |
| arRayK25/ctOases\_cat\_cd | -1.3158438 | -1.1728035082 | -0.52932276 | 1 |
| arRayK25/ctOases\_cat\_cd\_rcMin2 | -2.2740630 | -0.6793506720 | 0.20854386 | 1 |
| arRayK25/ctRayK25 | -2.3286980 | -0.5894490588 | 0.26692524 | 1 |
| arRayK25/ctRayK35 | -2.7351367 | -0.3421722652 | 0.65503564 | 1 |
| arRayK25/ctRay\_cat\_cd | -2.1996797 | -0.6090135360 | 0.17250875 | 1 |
| arRayK25/ctRay\_cat\_cd\_rcMin2 | -2.3734360 | -0.5869553664 | 0.29178484 | 1 |
| arRayK25/ctSoapK25 | -2.2039264 | -0.6467747746 | 0.17892395 | 1 |
| arRayK25/ctSoapK35 | -2.3265338 | -0.6164179319 | 0.27910683 | 1 |
| arRayK25/ctSoap\_cat\_cd | -1.3801591 | -0.9886811496 | -0.43737786 | 1 |
| arRayK25/ctSoap\_cat\_cd\_rcMin2 | -2.2655442 | -0.6831667147 | 0.22551227 | 1 |
| arRayK25/scOasesK25 | -0.8420050 | -0.4867463201 | -0.33188499 | 1 |
| arRayK25/scOasesK35 | -2.0833391 | -0.5274670099 | 0.29840818 | 1 |
| arRayK25/scOases\_cat\_cd | -0.1844525 | -0.8338473805 | -0.65842434 | 1 |
| arRayK25/scOases\_cat\_cd\_rcMin2 | -1.2682083 | -0.5921029278 | -0.10011547 | 1 |
| arRayK25/scRayK25 | -2.3481173 | -0.5860365000 | 0.25680946 | 1 |
| arRayK25/scRayK35 | -2.7362723 | -0.3295047194 | 0.67287987 | 1 |
| arRayK25/scRay\_cat\_cd | -2.2153004 | -0.5984947887 | 0.14289396 | 1 |
| arRayK25/scRay\_cat\_cd\_rcMin2 | -2.3818291 | -0.5796102422 | 0.27364859 | 1 |
| arRayK25/scSoapK25 | -2.2543545 | -0.6731846977 | 0.21115305 | 1 |
| arRayK25/scSoapK35 | -2.3689676 | -0.6098118594 | 0.32672457 | 1 |
| arRayK25/scSoap\_cat\_cd | -1.3683314 | -0.9752793891 | -0.36249805 | 1 |
| arRayK25/scSoap\_cat\_cd\_rcMin2 | -2.2711251 | -0.6919269140 | 0.22482853 | 1 |
| arRayK35/ctOasesK25 | -2.3494168 | -0.2638849246 | 0.21011568 | 1 |
| arRayK35/ctOasesK35 | -2.3949680 | -0.2356768229 | 0.21698727 | 1 |
| arRayK35/ctOases\_cat\_cd | -1.4914843 | -0.6161588808 | -0.49298434 | 1 |
| arRayK35/ctOases\_cat\_cd\_rcMin2 | -2.2273518 | -0.2692679614 | 0.07321019 | 1 |
| arRayK35/ctRayK25 | -2.2928506 | -0.1602887846 | 0.20749789 | 1 |
| arRayK35/ctRayK35 | -2.7707886 | 0.0436863287 | 0.61063940 | 1 |
| arRayK35/ctRay\_cat\_cd | -2.2160745 | -0.1799774874 | 0.11907065 | 1 |
| arRayK35/ctRay\_cat\_cd\_rcMin2 | -2.3765643 | -0.1574254821 | 0.21357269 | 1 |
| arRayK35/ctSoapK25 | -2.2011157 | -0.1876006440 | 0.08828101 | 1 |
| arRayK35/ctSoapK35 | -2.3648316 | -0.1602190783 | 0.30150749 | 1 |
| arRayK35/ctSoap\_cat\_cd | -1.3650594 | -0.4622860753 | -0.50915542 | 1 |
| arRayK35/ctSoap\_cat\_cd\_rcMin2 | -2.2168942 | -0.2391486742 | 0.05438824 | 1 |
| arRayK35/scOasesK25 | -0.7702461 | 0.0070265701 | -0.49939429 | 1 |
| arRayK35/scOasesK35 | -2.0388509 | -0.1140675024 | 0.20630167 | 1 |
| arRayK35/scOases\_cat\_cd | -0.1380392 | -0.2163232293 | -0.72371613 | 1 |
| arRayK35/scOases\_cat\_cd\_rcMin2 | -1.3038461 | -0.1557311646 | -0.38352385 | 1 |
| arRayK35/scRayK25 | -2.2787919 | -0.1551478025 | 0.19779252 | 1 |
| arRayK35/scRayK35 | -2.7138456 | 0.0847664144 | 0.67669860 | 1 |
| arRayK35/scRay\_cat\_cd | -2.2077382 | -0.1821971931 | 0.10367409 | 1 |
| arRayK35/scRay\_cat\_cd\_rcMin2 | -2.3575969 | -0.1612006136 | 0.18802773 | 1 |
| arRayK35/scSoapK25 | -2.2683985 | -0.2260351519 | 0.13472088 | 1 |
| arRayK35/scSoapK35 | -2.3984210 | -0.2106770794 | 0.24319745 | 1 |
| arRayK35/scSoap\_cat\_cd | -1.3660933 | -0.5157512815 | -0.49416564 | 1 |
| arRayK35/scSoap\_cat\_cd\_rcMin2 | -2.2450083 | -0.2465036231 | 0.08548717 | 1 |
| ctEulK29 | -2.1104375 | 2.7291602515 | 0.68243549 | 1 |
| ctRayK35 | -2.6288185 | 0.3470620079 | 0.28676869 | 1 |
| rrRayK35 | -1.5944227 | 0.7327353144 | -0.10661701 | 1 |
| scRayK35 | -2.6278883 | 0.3468002795 | 0.28605349 | 1 |
| aaMin2/ctRayK35/454Cap3 | 0.4766818 | 1.5940800528 | 0.81425616 | 2 |
| aaMin2/scRayK35/454Cap3 | 0.4772256 | 1.5938701448 | 0.81344635 | 2 |
| arMIRA/ctOasesK25 | 1.7384078 | 3.7778086642 | -0.37417074 | 2 |
| arMIRA/ctOasesK35 | 1.2011666 | 3.1400862273 | -0.35323773 | 2 |
| arMIRA/ctOases\_cat\_cd | 2.7890815 | 2.4702647071 | -1.12406459 | 2 |
| arMIRA/ctOases\_cat\_cd\_rcMin2 | 2.1848387 | 4.4641283071 | -0.67796911 | 2 |
| arMIRA/ctRayK25 | 1.6688318 | 4.1283661124 | -0.46923882 | 2 |
| arMIRA/ctRayK35 | 0.3592780 | 3.1996446284 | 0.36700226 | 2 |
| arMIRA/ctRay\_cat\_cd | 1.6921921 | 4.0651565940 | -0.56786951 | 2 |
| arMIRA/ctRay\_cat\_cd\_rcMin2 | 1.5741680 | 4.2565615231 | -0.52241537 | 2 |
| arMIRA/ctSoapK25 | 2.2062510 | 4.5761585610 | -0.66374009 | 2 |
| arMIRA/ctSoapK35 | 1.3815702 | 3.4291785208 | -0.35530273 | 2 |
| arMIRA/ctSoap\_cat\_cd | 2.9063391 | 3.3622717252 | -1.00247825 | 2 |
| arMIRA/ctSoap\_cat\_cd\_rcMin2 | 2.4758094 | 5.0233067482 | -0.64865819 | 2 |
| arMIRA/scOasesK25 | 3.5115014 | 5.0979665466 | -0.38883051 | 2 |
| arMIRA/scOasesK35 | 1.7881525 | 3.7078307583 | -0.30237228 | 2 |
| arMIRA/scOases\_cat\_cd | 4.4118473 | 4.3131011038 | -0.38350502 | 2 |
| arMIRA/scOases\_cat\_cd\_rcMin2 | 3.9053722 | 5.9142877980 | 0.13033479 | 2 |
| arMIRA/scRayK25 | 1.5449129 | 4.0495724988 | -0.59028426 | 2 |
| arMIRA/scRayK35 | 0.3606334 | 3.1987665970 | 0.36396182 | 2 |
| arMIRA/scRay\_cat\_cd | 1.6308072 | 4.0238033207 | -0.62894682 | 2 |
| arMIRA/scRay\_cat\_cd\_rcMin2 | 1.6339310 | 4.3064678858 | -0.45232452 | 2 |
| arMIRA/scSoapK25 | 2.1763329 | 4.5659974431 | -0.68763280 | 2 |
| arMIRA/scSoapK35 | 1.3231117 | 3.3829521876 | -0.42506363 | 2 |
| arMIRA/scSoap\_cat\_cd | 2.8737455 | 3.3480275089 | -1.04983083 | 2 |
| arMIRA/scSoap\_cat\_cd\_rcMin2 | 2.4817946 | 5.0156246067 | -0.63197821 | 2 |
| ctMIRA | -0.7006531 | 3.0896674891 | 2.07911219 | 2 |
| ctMIRA\_ctEulK29\_rcCAP3 | -0.1674574 | 2.9406828339 | 1.87796085 | 2 |
| aaMin2/ctALL/454Cap3 | 4.4386147 | -1.7311243816 | -0.55031549 | 3 |
| aaMin2/ctOasesK25/454Cap3 | 3.1714640 | -1.8237584776 | -0.81183771 | 3 |
| aaMin2/ctOasesK35/454Cap3 | 2.2082809 | -1.3545859455 | -0.29585880 | 3 |
| aaMin2/ctOases\_cat\_cd/454Cap3 | 4.0208266 | -1.5152335349 | -0.62563801 | 3 |
| aaMin2/ctOases\_cat\_cd\_rcMin2/454Cap3 | 4.1012249 | -1.5795450775 | -0.65132458 | 3 |
| aaMin2/ctRayK25/454Cap3 | 2.3621300 | -0.0729629998 | -0.05956485 | 3 |
| aaMin2/ctRay\_cat\_cd/454Cap3 | 2.3160624 | -0.0609699211 | -0.17946165 | 3 |
| aaMin2/ctRay\_cat\_cd\_rcMin2/454Cap3 | 2.3843876 | -0.0480703767 | -0.11553724 | 3 |
| aaMin2/ctSoapK25/454Cap3 | 3.9801690 | -2.3777533493 | -0.79142262 | 3 |
| aaMin2/ctSoapK35/454Cap3 | 2.2025180 | -1.2234414537 | -0.22585923 | 3 |
| aaMin2/ctSoap\_cat\_cd/454Cap3 | 4.3995470 | -2.0266362444 | -0.59325939 | 3 |
| aaMin2/ctSoap\_cat\_cd\_rcMin2/454Cap3 | 4.4748074 | -2.1002959305 | -0.61005306 | 3 |
| aaMin2/scOasesK35/454Cap3 | 2.9521428 | -0.7935046876 | 0.03574897 | 3 |
| aaMin2/scRayK25/454Cap3 | 2.3170427 | -0.1076439453 | -0.11287699 | 3 |
| aaMin2/scRay\_cat\_cd/454Cap3 | 2.3419110 | -0.0447913419 | -0.15460008 | 3 |
| aaMin2/scRay\_cat\_cd\_rcMin2/454Cap3 | 2.4087133 | -0.0318562098 | -0.08924265 | 3 |
| aaMin2/scSoapK25/454Cap3 | 3.6386574 | -1.3236130823 | -0.71465039 | 3 |
| aaMin2/scSoapK35/454Cap3 | 2.2334624 | -1.1359895256 | -0.17390088 | 3 |
| aaMin2/scSoap\_cat\_cd/454Cap3 | 4.1466006 | -1.0336941342 | -0.52926689 | 3 |
| aaMin2/scSoap\_cat\_cd\_rcMin2/454Cap3 | 4.1863246 | -1.1152031478 | -0.55640952 | 3 |
| ctOasesK25 | 1.3503496 | -2.2428469036 | -2.09438106 | 3 |
| ctOasesK35 | 0.1508794 | -1.8589688250 | -1.42075336 | 3 |
| ctOases\_cat | 3.6408831 | -3.5695944707 | -1.97562432 | 3 |
| ctOases\_cat\_cd | 2.7907406 | -2.7821298343 | -1.89691931 | 3 |
| ctOases\_cat\_cd\_rcMin2 | 2.9452401 | -2.1350104191 | -1.12334229 | 3 |
| ctRayK25 | 0.4329573 | -0.8089900940 | -1.33241631 | 3 |
| ctRay\_cat | 1.1624721 | -1.1007379593 | -0.97192075 | 3 |
| ctRay\_cat\_cd | 0.6806379 | -0.8084138127 | -1.20827608 | 3 |
| ctRay\_cat\_cd\_rcMin2 | 0.9222762 | -0.6963497299 | -0.51782117 | 3 |
| ctSoapK25 | 2.3346695 | -2.6711921635 | -2.06464917 | 3 |
| ctSoapK35 | 0.3157897 | -1.7217246035 | -1.40656849 | 3 |
| ctSoap\_cat | 4.9244331 | -3.9724617922 | -1.16796807 | 3 |
| ctSoap\_cat\_cd | 3.5732722 | -2.9984943217 | -1.60153529 | 3 |
| ctSoap\_cat\_cd\_rcMin2 | 3.5114642 | -2.5008169527 | -1.00623166 | 3 |
| rrRayK25 | 0.8937263 | -0.5659613295 | -1.37014316 | 3 |
| scOasesK35 | 1.9199421 | -1.6571519125 | 1.04008201 | 3 |
| scRayK25 | 0.4546665 | -0.7912704934 | -1.30458610 | 3 |
| scRay\_cat | 1.1772851 | -1.0883187310 | -0.95245054 | 3 |
| scRay\_cat\_cd | 0.6792030 | -0.8078225519 | -1.20704712 | 3 |
| scRay\_cat\_cd\_rcMin2 | 0.9224462 | -0.6962757299 | -0.51657663 | 3 |
| scSoapK25 | 2.0831784 | -1.6123810975 | -1.91203592 | 3 |
| scSoapK35 | 0.2989923 | -1.6332871746 | -1.39516690 | 3 |
| scSoap\_cat | 4.5656244 | -3.1001476739 | -0.96878344 | 3 |
| scSoap\_cat\_cd | 3.2984827 | -2.0975544869 | -1.44647859 | 3 |
| scSoap\_cat\_cd\_rcMin2 | 3.1484111 | -1.5555310540 | -0.95406745 | 3 |
| A.thaliana | 11.6123460 | 8.8373272812 | 1.18998480 | 4 |
| aaMin2/scALL/454Cap3 | 7.4094369 | -0.1426689144 | 1.72637223 | 4 |
| aaMin2/scOasesK25/454Cap3 | 6.7290771 | -0.2352255326 | 1.17048081 | 4 |
| aaMin2/scOases\_cat\_cd/454Cap3 | 7.3818239 | -0.2265952735 | 1.35751031 | 4 |
| aaMin2/scOases\_cat\_cd\_rcMin2/454Cap3 | 7.6018401 | -0.2222310909 | 1.42794469 | 4 |
| scOasesK25 | 7.3557186 | -1.5342861011 | 4.88595732 | 4 |
| scOases\_cat | 9.7521384 | -3.3705452145 | 5.80873332 | 4 |
| scOases\_cat\_cd | 8.6896485 | -2.3805669841 | 5.39690981 | 4 |
| scOases\_cat\_cd\_rcMin2 | 6.7772957 | -0.5157392338 | 1.33430672 | 4 |

| PCA dimension 1 | | |
| --- | --- | --- |
| **Variables** | | |
| *Name* | *Correlation coef* | *p-valor* |
| Contigs500 | 0.9814147 | 2.837311e-130 |
| ComplOrtho | 0.9550712 | 1.797515e-96 |
| DiffProts | 0.9370483 | 1.030486e-83 |
| DiffComplProts | 0.9219851 | 1.135755e-75 |
| AllTransSize | 0.9200494 | 9.325234e-75 |
| DuplOrtho | 0.7684131 | 1.542793e-36 |
| MissAssembl | 0.7441387 | 3.461402e-33 |
| FragOrtho | 0.7417458 | 7.064589e-33 |
| MeanContigCov | 0.7255300 | 7.277481e-31 |
| Contigs | 0.7087978 | 6.212675e-29 |
| Ns | 0.6176021 | 2.039581e-20 |
| MeanGapLen | 0.6146439 | 3.467031e-20 |
| N50 | 0.5639545 | 1.379797e-16 |
| MeanContigLen | 0.3571582 | 7.992592e-07 |
| N90 | 0.1850275 | 1.264502e-02 |
| **Factors** | | |
| *Name* | *R2* | *p-valor* |
| Program | 0.7740278 | 1.494920e-51 |
| Kmer | 0.6302262 | 2.018612e-33 |
| Technology | 0.2012247 | 1.131129e-08 |
| Task | 0.2377623 | 3.377728e-07 |
| **Categories** | | |
| *Name* | *Estimate* | *p-valor* |
| minimus | 2.1307996 | 1.800168e-16 |
| 25;35 | 2.3496154 | 7.969876e-12 |
| 29/25;35 | 3.6264185 | 6.127927e-10 |
| Ill | 0.7751970 | 1.103697e-07 |
| join | 1.9008754 | 2.117023e-06 |
| mira | 0.5771982 | 2.219806e-04 |
| cat | 2.8162273 | 2.593253e-04 |
| 29/25 | 2.9253094 | 1.368968e-03 |
| 454\_Ill | 4.2124764 | 3.511453e-03 |
| cd-hit | 1.8977521 | 4.599749e-03 |
| merge | 1.1941625 | 8.896204e-03 |
| SC\_trs\_all | 5.5657439 | 1.024289e-02 |
| rs\_454read/Ill | -2.4952841 | 1.388682e-02 |
| 25 | -2.0179426 | 2.856827e-05 |
| 454-Ill | -2.2832747 | 1.925992e-08 |
| 35 | -2.5654488 | 5.580484e-10 |
| Ray | -3.5461471 | 1.084265e-50 |

| PCA dimension 2 | | |
| --- | --- | --- |
| **Variables** | | |
| *Name* | *Correlation coef* | *p-valor* |
| N90 | 0.9492043 | 8.148236e-92 |
| MeanContigLen | 0.9173712 | 1.575310e-73 |
| N50 | 0.8092427 | 3.270355e-43 |
| MissAssembl | 0.3427440 | 2.323293e-06 |
| MeanContigCov | 0.2759878 | 1.694958e-04 |
| DiffComplProts | 0.2424340 | 1.008255e-03 |
| DiffProts | -0.1745352 | 1.877702e-02 |
| DuplOrtho | -0.1815334 | 1.445640e-02 |
| Ns | -0.2139708 | 3.824167e-03 |
| MeanGapLen | -0.2426300 | 9.984858e-04 |
| FragOrtho | -0.3078877 | 2.482438e-05 |
| AllTransSize | -0.3428875 | 2.299340e-06 |
| Contigs | -0.6099512 | 7.953113e-20 |
| **Factors** | | |
| *Name* | *R2* | *p-valor* |
| Program | 0.8851475 | 1.175730e-76 |
| Task | 0.3104790 | 1.560542e-10 |
| Technology | 0.2131599 | 3.067352e-09 |
| **Categories** | | |
| *Name* | *Estimate* | *p-valor* |
| mira | 3.9900453 | 6.030792e-56 |
| rs\_454read/Ill | 1.4030953 | 3.952664e-11 |
| 454-Ill | 0.1695484 | 2.450652e-06 |
| 454 | 2.7802823 | 5.551169e-03 |
| oases | -1.8295480 | 4.605061e-02 |
| SOAPdenovo | -1.9158809 | 3.657048e-02 |
| rs | -0.0878010 | 1.011141e-02 |
| cd-hit | -1.9853983 | 7.380925e-03 |
| Ray | -0.3525978 | 3.954615e-03 |
| minimus | -0.8574586 | 3.869478e-03 |
| cat | -2.7065356 | 2.259643e-04 |
| join | -1.9943231 | 3.321012e-06 |
| Ill | -1.8733795 | 4.487043e-09 |
