# Supplementary material for: TransFlow: a modular framework for assembling and assessing accurate de novo transcriptomes in non-model organisms
Source: BMC Bioinformatics. 2018 Nov 20;19(Suppl 14):416. doi: 10.1186/s12859-018-2384-y (PMC6245506; doi:10.1186/s12859-018-2384-y)
Supplement: Supplementary file 4 — HTML report of TransFlow for P. xanthii. The zip file contains the elements of the report: the HTML file called assembly_report.html that can be open in any browser (javascript must be enabled) and inspected thoroughly; the folder js must be side-by-side to the HTML file for the right function. (ZIP 889 kb) [file 12859_2018_2384_MOESM4_ESM.zip › assembly_report.html]

assembly\_report


| PCA Ranking | |
| --- | --- |
| **Name** | **PCA distance** |
| arMIRA/scSoap\_cat\_cd\_rcMin2 | 0.04701257 |
| arMIRA/ctSoapK25 | 0.04730009 |
| arMIRA/scRay\_cat\_cd\_rcMin2 | 0.04763557 |
| arMIRA/ctRayK35 | 0.04796294 |
| arMIRA/ctSoap\_cat\_cd\_rcMin2 | 0.04798089 |
| arMIRA/scOases\_cat\_cd\_rcMin2 | 0.04800336 |
| arMIRA/scSoapK35 | 0.04804746 |
| arMIRA/scRayK35 | 0.04806140 |
| arMIRA/ctRayK25 | 0.04819116 |
| arMIRA/scSoapK25 | 0.04820887 |
| arMIRA/scOasesK25 | 0.04833889 |
| arMIRA/scOasesK35 | 0.04844761 |
| arMIRA/scRayK25 | 0.04880550 |
| arMIRA/ctSoapK35 | 0.04881848 |
| arMIRA/ctRay\_cat\_cd\_rcMin2 | 0.04888435 |
| arMIRA/ctRay\_cat\_cd | 0.04921724 |
| arMIRA/scRay\_cat\_cd | 0.04984005 |
| arMIRA/ctOasesK35 | 0.05036611 |
| arMIRA/ctOasesK25 | 0.05047266 |
| arMIRA/ctOases\_cat\_cd\_rcMin2 | 0.05069596 |
| ctMIRA\_ctEulK29\_rcCAP3 | 0.05587010 |
| arMIRA/scOases\_cat\_cd | 0.05932741 |
| arMIRA/ctOases\_cat\_cd | 0.06232902 |
| arMIRA/scSoap\_cat\_cd | 0.06823820 |
| ctMIRA | 0.06886271 |
| arMIRA/ctSoap\_cat\_cd | 0.06958832 |
| ctEulK29 | 0.14351849 |
| aaMin2/scOasesK35/454Cap3 | 0.16145719 |
| aaMin2/ctRayK35/454Cap3 | 0.16268392 |
| aaMin2/ctOasesK35/454Cap3 | 0.16367867 |
| aaMin2/scRayK35/454Cap3 | 0.16425391 |
| aaMin2/ctOasesK25/454Cap3 | 0.17140907 |
| aaMin2/scOasesK25/454Cap3 | 0.17757310 |
| aaMin2/ctOases\_cat\_cd/454Cap3 | 0.18348114 |
| aaMin2/ctOases\_cat\_cd\_rcMin2/454Cap3 | 0.18402763 |
| arRayK35/ctRay\_cat\_cd\_rcMin2 | 0.19164527 |
| arRayK35/scRay\_cat\_cd\_rcMin2 | 0.19204051 |
| arRayK35/scRayK25 | 0.19230009 |
| arRayK35/ctRayK25 | 0.19238937 |
| arRayK35/ctSoap\_cat\_cd\_rcMin2 | 0.19268467 |
| arRayK35/scOasesK35 | 0.19278015 |
| arRayK35/scOasesK25 | 0.19283053 |
| arRayK35/scRay\_cat\_cd | 0.19303307 |
| arRayK35/ctOases\_cat\_cd\_rcMin2 | 0.19329737 |
| arRayK35/ctRay\_cat\_cd | 0.19339458 |
| arRayK35/scRayK35 | 0.19350038 |
| arRayK35/scSoap\_cat\_cd\_rcMin2 | 0.19357019 |
| arRayK35/ctOasesK35 | 0.19358026 |
| arRayK35/ctSoapK25 | 0.19360385 |
| arRayK35/scSoapK35 | 0.19371117 |
| arRayK35/ctOasesK25 | 0.19375713 |
| aaMin2/scOases\_cat\_cd/454Cap3 | 0.19382194 |
| arRayK35/scOases\_cat\_cd\_rcMin2 | 0.19430801 |
| arRayK35/scSoapK25 | 0.19476232 |
| arRayK35/ctRayK35 | 0.19492530 |
| arRayK35/ctSoapK35 | 0.19543412 |
| arRayK35/scOases\_cat\_cd | 0.19650570 |
| aaMin2/scOases\_cat\_cd\_rcMin2/454Cap3 | 0.19690096 |
| arRayK35/ctOases\_cat\_cd | 0.19856229 |
| arRayK35/ctSoap\_cat\_cd | 0.20059214 |
| arRayK35/scSoap\_cat\_cd | 0.20242364 |
| arRayK25/ctOasesK25 | 0.21066490 |
| aaMin2/ctRayK25/454Cap3 | 0.21066710 |
| arRayK25/ctOases\_cat\_cd\_rcMin2 | 0.21136676 |
| arRayK25/scOasesK25 | 0.21164820 |
| arRayK25/scRay\_cat\_cd\_rcMin2 | 0.21178453 |
| arRayK25/ctRay\_cat\_cd\_rcMin2 | 0.21185945 |
| aaMin2/ctRay\_cat\_cd\_rcMin2/454Cap3 | 0.21192966 |
| arRayK25/ctOasesK35 | 0.21207729 |
| aaMin2/ctRay\_cat\_cd/454Cap3 | 0.21209256 |
| arRayK25/scRayK35 | 0.21219942 |
| arRayK25/scOasesK35 | 0.21237539 |
| arRayK25/scOases\_cat\_cd\_rcMin2 | 0.21254084 |
| arRayK25/scSoapK35 | 0.21267230 |
| arRayK25/ctRayK35 | 0.21275888 |
| arRayK25/ctRay\_cat\_cd | 0.21279200 |
| arRayK25/scRay\_cat\_cd | 0.21299905 |
| aaMin2/scRayK25/454Cap3 | 0.21315600 |
| arRayK25/scRayK25 | 0.21327780 |
| arRayK25/ctRayK25 | 0.21332424 |
| aaMin2/scRay\_cat\_cd/454Cap3 | 0.21364391 |
| arRayK25/scSoap\_cat\_cd\_rcMin2 | 0.21394111 |
| aaMin2/scRay\_cat\_cd\_rcMin2/454Cap3 | 0.21399443 |
| arRayK25/scSoapK25 | 0.21434334 |
| arRayK25/ctSoapK35 | 0.21443586 |
| arRayK25/ctSoap\_cat\_cd\_rcMin2 | 0.21470998 |
| arRayK25/ctSoapK25 | 0.21528485 |
| aaMin2/ctSoapK35/454Cap3 | 0.21646414 |
| arRayK25/scOases\_cat\_cd | 0.21857201 |
| arRayK25/ctOases\_cat\_cd | 0.21914792 |
| aaMin2/scSoapK35/454Cap3 | 0.22277904 |
| arRayK25/scSoap\_cat\_cd | 0.22526985 |
| arRayK25/ctSoap\_cat\_cd | 0.22573709 |
| aaMin2/scSoapK25/454Cap3 | 0.22877714 |
| aaMin2/scSoap\_cat\_cd/454Cap3 | 0.24542753 |
| aaMin2/ctSoapK25/454Cap3 | 0.24593834 |
| aaMin2/scSoap\_cat\_cd\_rcMin2/454Cap3 | 0.24641481 |
| aaMin2/ctSoap\_cat\_cd/454Cap3 | 0.25187928 |
| aaMin2/ctSoap\_cat\_cd\_rcMin2/454Cap3 | 0.25271765 |
| aaMin2/ctALL/454Cap3 | 0.25410655 |
| aaMin2/scALL/454Cap3 | 0.27631340 |
| rrRayK35 | 0.31324369 |
| rrRayK25 | 0.32644364 |
| aaRayK35/ctSoapK25/454Cap3 | 0.33349156 |
| aaRayK35/ctSoap\_cat\_cd\_rcMin2/454Cap3 | 0.33586077 |
| aaRayK35/scSoap\_cat\_cd\_rcMin2/454Cap3 | 0.33986782 |
| aaRayK35/ctSoap\_cat\_cd/454Cap3 | 0.34018018 |
| aaRayK35/scSoapK25/454Cap3 | 0.34055580 |
| aaRayK35/scSoapK35/454Cap3 | 0.34130464 |
| aaRayK35/scRay\_cat\_cd/454Cap3 | 0.34151080 |
| aaRayK35/ctSoapK35/454Cap3 | 0.34260639 |
| aaRayK35/scOasesK25/454Cap3 | 0.34272119 |
| aaRayK35/scOases\_cat\_cd/454Cap3 | 0.34273541 |
| aaRayK35/ctRay\_cat\_cd/454Cap3 | 0.34354311 |
| aaRayK35/scRayK25/454Cap3 | 0.34378903 |
| aaRayK35/scRay\_cat\_cd\_rcMin2/454Cap3 | 0.34421177 |
| aaRayK35/scSoap\_cat\_cd/454Cap3 | 0.34446544 |
| aaRayK35/ctRayK25/454Cap3 | 0.34565482 |
| aaRayK35/scOases\_cat\_cd\_rcMin2/454Cap3 | 0.34660370 |
| aaRayK35/ctRay\_cat\_cd\_rcMin2/454Cap3 | 0.34665966 |
| aaRayK35/scOasesK35/454Cap3 | 0.34849476 |
| aaRayK35/scRayK35/454Cap3 | 0.35364768 |
| aaRayK35/ctRayK35/454Cap3 | 0.35471065 |
| aaRayK35/ctOases\_cat\_cd/454Cap3 | 0.36022927 |
| aaRayK35/ctOasesK25/454Cap3 | 0.36054657 |
| aaRayK35/ctOases\_cat\_cd\_rcMin2/454Cap3 | 0.36055411 |
| aaRayK35/ctOasesK35/454Cap3 | 0.36550840 |
| aaRayK25/ctSoapK25/454Cap3 | 0.36561724 |
| aaRayK25/scSoap\_cat\_cd\_rcMin2/454Cap3 | 0.36750038 |
| aaRayK25/ctSoap\_cat\_cd\_rcMin2/454Cap3 | 0.36910539 |
| aaRayK25/scSoapK25/454Cap3 | 0.36941031 |
| aaRayK25/ctSoapK35/454Cap3 | 0.37051770 |
| aaRayK25/scRayK35/454Cap3 | 0.37056721 |
| aaRayK25/scSoapK35/454Cap3 | 0.37071226 |
| aaRayK25/ctRay\_cat\_cd/454Cap3 | 0.37237583 |
| aaRayK25/ctRay\_cat\_cd\_rcMin2/454Cap3 | 0.37336436 |
| aaRayK25/scOasesK25/454Cap3 | 0.37368292 |
| aaRayK25/ctRayK35/454Cap3 | 0.37397436 |
| aaRayK25/scRay\_cat\_cd\_rcMin2/454Cap3 | 0.37500639 |
| aaRayK25/ctSoap\_cat\_cd/454Cap3 | 0.37630297 |
| aaRayK25/scOases\_cat\_cd\_rcMin2/454Cap3 | 0.37667529 |
| aaRayK25/scRayK25/454Cap3 | 0.37703329 |
| aaRayK25/scRay\_cat\_cd/454Cap3 | 0.37785634 |
| aaRayK25/ctRayK25/454Cap3 | 0.37785727 |
| aaRayK25/scOases\_cat\_cd/454Cap3 | 0.37845153 |
| aaRayK25/scOasesK35/454Cap3 | 0.37930490 |
| aaRayK25/scSoap\_cat\_cd/454Cap3 | 0.38151253 |
| scOases\_cat\_cd\_rcMin2 | 0.38483729 |
| scOases\_cat | 0.38500695 |
| scOases\_cat\_cd | 0.38619639 |
| aaRayK25/ctOases\_cat\_cd\_rcMin2/454Cap3 | 0.38770355 |
| scSoap\_cat\_cd | 0.38870523 |
| aaRayK25/ctOasesK35/454Cap3 | 0.39021448 |
| aaRayK25/ctOases\_cat\_cd/454Cap3 | 0.39079635 |
| scOasesK35 | 0.39158064 |
| aaRayK25/ctOasesK25/454Cap3 | 0.39222812 |
| scSoapK35 | 0.39569371 |
| ctSoapK35 | 0.39660207 |
| scSoap\_cat\_cd\_rcMin2 | 0.39697112 |
| ctSoap\_cat\_cd | 0.39881878 |
| scOasesK25 | 0.39898013 |
| scSoapK25 | 0.39943667 |
| ctRayK35 | 0.40334537 |
| ctSoap\_cat\_cd\_rcMin2 | 0.40589034 |
| ctSoap\_cat | 0.40924350 |
| scSoap\_cat | 0.40937103 |
| ctOases\_cat | 0.40999918 |
| ctRay\_cat\_cd | 0.41053405 |
| ctOases\_cat\_cd | 0.41129198 |
| ctRay\_cat\_cd\_rcMin2 | 0.41170391 |
| ctRay\_cat | 0.41183727 |
| ctSoapK25 | 0.41329378 |
| ctOases\_cat\_cd\_rcMin2 | 0.41431937 |
| ctOasesK35 | 0.41496745 |
| scRay\_cat\_cd\_rcMin2 | 0.41527776 |
| ctOasesK25 | 0.42314642 |
| ctRayK25 | 0.42935204 |
| scRayK35 | 0.44778270 |
| scRay\_cat\_cd | 0.45387204 |
| scRay\_cat | 0.46917538 |
| scRayK25 | 0.47979081 |

| Cluster data | | | | |
| --- | --- | --- | --- | --- |
| **Name** | **Coord Dim1** | **Coord Dim2** | **Coord Dim3** | **Cluster** |
| aaRayK25/ctOasesK25/454Cap3 | -3.2391348 | -1.07909754 | -0.39744493 | 1 |
| aaRayK25/ctOasesK35/454Cap3 | -3.2094663 | -1.12834606 | -0.40439697 | 1 |
| aaRayK25/ctOases\_cat\_cd/454Cap3 | -3.1938434 | -0.99183188 | -0.44866854 | 1 |
| aaRayK25/ctOases\_cat\_cd\_rcMin2/454Cap3 | -3.1623454 | -1.06481991 | -0.40509472 | 1 |
| aaRayK25/ctRayK25/454Cap3 | -2.9954929 | -1.06347257 | -0.42507140 | 1 |
| aaRayK25/ctRayK35/454Cap3 | -2.9465303 | -1.20726116 | -0.41867703 | 1 |
| aaRayK25/ctRay\_cat\_cd/454Cap3 | -2.8981050 | -1.03586847 | -0.43846669 | 1 |
| aaRayK25/ctRay\_cat\_cd\_rcMin2/454Cap3 | -2.9150370 | -1.06755536 | -0.44899613 | 1 |
| aaRayK25/ctSoapK25/454Cap3 | -2.7921042 | -1.04167682 | -0.41360856 | 1 |
| aaRayK25/ctSoapK35/454Cap3 | -2.8769423 | -1.03720486 | -0.40185163 | 1 |
| aaRayK25/ctSoap\_cat\_cd/454Cap3 | -2.9301743 | -0.77934443 | -0.45153150 | 1 |
| aaRayK25/ctSoap\_cat\_cd\_rcMin2/454Cap3 | -2.8541565 | -1.02257466 | -0.39282821 | 1 |
| aaRayK25/scOasesK25/454Cap3 | -2.9796856 | -0.99876277 | -0.18750695 | 1 |
| aaRayK25/scOasesK35/454Cap3 | -3.0429462 | -1.15368090 | -0.36805092 | 1 |
| aaRayK25/scOases\_cat\_cd/454Cap3 | -3.0319480 | -0.86395301 | -0.23359178 | 1 |
| aaRayK25/scOases\_cat\_cd\_rcMin2/454Cap3 | -2.9933258 | -1.09982104 | -0.37234793 | 1 |
| aaRayK25/scRayK25/454Cap3 | -2.9786008 | -1.06462272 | -0.43788180 | 1 |
| aaRayK25/scRayK35/454Cap3 | -2.8805219 | -1.20261076 | -0.45199508 | 1 |
| aaRayK25/scRay\_cat\_cd/454Cap3 | -2.9985684 | -1.02642508 | -0.39869013 | 1 |
| aaRayK25/scRay\_cat\_cd\_rcMin2/454Cap3 | -2.9458924 | -1.06550117 | -0.43484925 | 1 |
| aaRayK25/scSoapK25/454Cap3 | -2.8602644 | -1.02653653 | -0.39061121 | 1 |
| aaRayK25/scSoapK35/454Cap3 | -2.8800639 | -1.01902247 | -0.39504536 | 1 |
| aaRayK25/scSoap\_cat\_cd/454Cap3 | -3.0224439 | -0.74957311 | -0.41426566 | 1 |
| aaRayK25/scSoap\_cat\_cd\_rcMin2/454Cap3 | -2.8255620 | -1.01303673 | -0.39536118 | 1 |
| aaRayK35/ctOasesK25/454Cap3 | -2.7863267 | -1.40419857 | -0.22989778 | 1 |
| aaRayK35/ctOasesK35/454Cap3 | -2.8704607 | -1.40366199 | -0.22625047 | 1 |
| aaRayK35/ctOases\_cat\_cd/454Cap3 | -2.7703383 | -1.28773786 | -0.23639857 | 1 |
| aaRayK35/ctOases\_cat\_cd\_rcMin2/454Cap3 | -2.7893592 | -1.37663585 | -0.21014304 | 1 |
| aaRayK35/ctRayK25/454Cap3 | -2.5344110 | -1.43901735 | -0.23221084 | 1 |
| aaRayK35/ctRayK35/454Cap3 | -2.6894669 | -1.44338501 | -0.23335435 | 1 |
| aaRayK35/ctRay\_cat\_cd/454Cap3 | -2.4954715 | -1.37540067 | -0.22354455 | 1 |
| aaRayK35/ctRay\_cat\_cd\_rcMin2/454Cap3 | -2.5525567 | -1.41846203 | -0.22322873 | 1 |
| aaRayK35/ctSoapK25/454Cap3 | -2.3258065 | -1.40181176 | -0.20911248 | 1 |
| aaRayK35/ctSoapK35/454Cap3 | -2.4825027 | -1.36976017 | -0.20912798 | 1 |
| aaRayK35/ctSoap\_cat\_cd/454Cap3 | -2.4174651 | -1.21255120 | -0.24595878 | 1 |
| aaRayK35/ctSoap\_cat\_cd\_rcMin2/454Cap3 | -2.3662614 | -1.37540540 | -0.20572073 | 1 |
| aaRayK35/scOasesK25/454Cap3 | -2.5041302 | -1.44779059 | -0.15406687 | 1 |
| aaRayK35/scOasesK35/454Cap3 | -2.5964240 | -1.46232335 | -0.18656080 | 1 |
| aaRayK35/scOases\_cat\_cd/454Cap3 | -2.4861563 | -1.30838839 | -0.18419019 | 1 |
| aaRayK35/scOases\_cat\_cd\_rcMin2/454Cap3 | -2.5680646 | -1.41090843 | -0.15560680 | 1 |
| aaRayK35/scRayK25/454Cap3 | -2.4981461 | -1.43112577 | -0.24570589 | 1 |
| aaRayK35/scRayK35/454Cap3 | -2.6682155 | -1.43793591 | -0.24435342 | 1 |
| aaRayK35/scRay\_cat\_cd/454Cap3 | -2.4572983 | -1.37723065 | -0.23547562 | 1 |
| aaRayK35/scRay\_cat\_cd\_rcMin2/454Cap3 | -2.5073561 | -1.41674850 | -0.23432938 | 1 |
| aaRayK35/scSoapK25/454Cap3 | -2.4569616 | -1.36917144 | -0.16939163 | 1 |
| aaRayK35/scSoapK35/454Cap3 | -2.4559909 | -1.35283760 | -0.21927793 | 1 |
| aaRayK35/scSoap\_cat\_cd/454Cap3 | -2.4987725 | -1.13820124 | -0.19146003 | 1 |
| aaRayK35/scSoap\_cat\_cd\_rcMin2/454Cap3 | -2.4427689 | -1.33171892 | -0.16646086 | 1 |
| ctOasesK25 | -3.5490985 | 0.07390970 | -0.45297740 | 1 |
| ctOasesK35 | -3.4423000 | -0.04134401 | -0.45058716 | 1 |
| ctOases\_cat\_cd | -3.2934691 | 0.46998054 | -0.38292706 | 1 |
| ctOases\_cat\_cd\_rcMin2 | -3.3209845 | 0.59182974 | -0.35131309 | 1 |
| ctOases\_cat | -3.0889607 | 1.23401335 | -0.29334826 | 2 |
| ctRayK25 | -3.3549695 | 2.18066557 | 1.28978705 | 2 |
| ctRayK35 | -3.3125026 | 1.01903882 | 1.19128860 | 2 |
| ctRay\_cat | -2.5665384 | 3.32267464 | 1.41433179 | 2 |
| ctRay\_cat\_cd | -3.0644793 | 2.00429402 | 0.87088033 | 2 |
| ctRay\_cat\_cd\_rcMin2 | -3.1227220 | 1.76908740 | 0.57540058 | 2 |
| ctSoapK25 | -2.9141975 | 2.14614780 | 0.09154328 | 2 |
| ctSoapK35 | -2.8285099 | 1.46414479 | -0.14902944 | 2 |
| ctSoap\_cat | -1.6688046 | 4.44397102 | 0.27490125 | 2 |
| ctSoap\_cat\_cd | -2.4758012 | 2.59300200 | 0.07580489 | 2 |
| ctSoap\_cat\_cd\_rcMin2 | -2.6025240 | 2.48044091 | -0.11576564 | 2 |
| rrRayK25 | -1.2488872 | 2.91618659 | 3.21084508 | 2 |
| rrRayK35 | -1.3308981 | 2.09640555 | 3.84718208 | 2 |
| scOasesK25 | -2.8611552 | 2.32136699 | 2.14611699 | 2 |
| scOasesK35 | -3.0818773 | 1.22134800 | 1.37538470 | 2 |
| scOases\_cat | -1.9992848 | 3.54293969 | 1.70653807 | 2 |
| scOases\_cat\_cd | -2.3212657 | 3.02663991 | 2.16828679 | 2 |
| scOases\_cat\_cd\_rcMin2 | -2.5019998 | 2.24597876 | 0.65944301 | 2 |
| scRayK25 | -3.6811738 | 3.04562355 | 4.49480989 | 2 |
| scRayK35 | -3.6533140 | 1.85570797 | 4.48815738 | 2 |
| scRay\_cat | -2.8788152 | 4.23634271 | 4.60705470 | 2 |
| scRay\_cat\_cd | -3.3816147 | 2.94052052 | 4.09569260 | 2 |
| scRay\_cat\_cd\_rcMin2 | -3.1122092 | 2.00972230 | 0.68797781 | 2 |
| scSoapK25 | -2.7306005 | 2.12814857 | 0.29052640 | 2 |
| scSoapK35 | -2.7324574 | 1.83622387 | 0.06881212 | 2 |
| scSoap\_cat | -1.4398825 | 4.75298801 | 0.21616902 | 2 |
| scSoap\_cat\_cd | -2.1127918 | 2.96255818 | 0.04296999 | 2 |
| scSoap\_cat\_cd\_rcMin2 | -2.3594550 | 2.48816729 | -0.44045174 | 2 |
| arRayK25/ctOasesK25 | 0.3333658 | -1.13989409 | -0.68920759 | 3 |
| arRayK25/ctOasesK35 | 0.2873875 | -1.14536485 | -0.65982360 | 3 |
| arRayK25/ctOases\_cat\_cd | 0.1822087 | -0.99718098 | -0.75496027 | 3 |
| arRayK25/ctOases\_cat\_cd\_rcMin2 | 0.3191836 | -1.12600445 | -0.69043422 | 3 |
| arRayK25/ctRayK25 | 0.2780695 | -1.08684276 | -0.68922389 | 3 |
| arRayK25/ctRayK35 | 0.2635426 | -1.15906262 | -0.64481219 | 3 |
| arRayK25/ctRay\_cat\_cd | 0.3008083 | -1.03090475 | -0.69445764 | 3 |
| arRayK25/ctRay\_cat\_cd\_rcMin2 | 0.3133305 | -1.09385096 | -0.69362550 | 3 |
| arRayK25/ctSoapK25 | 0.2206923 | -1.10073212 | -0.66547671 | 3 |
| arRayK25/ctSoapK35 | 0.2498791 | -1.08536580 | -0.68341264 | 3 |
| arRayK25/ctSoap\_cat\_cd | 0.1157771 | -0.72093033 | -0.84999005 | 3 |
| arRayK25/ctSoap\_cat\_cd\_rcMin2 | 0.2500197 | -1.07545303 | -0.69609826 | 3 |
| arRayK25/scOasesK25 | 0.3220023 | -1.06592073 | -0.69291441 | 3 |
| arRayK25/scOasesK35 | 0.2852835 | -1.10171037 | -0.65721437 | 3 |
| arRayK25/scOases\_cat\_cd | 0.2202603 | -0.89305372 | -0.77497600 | 3 |
| arRayK25/scOases\_cat\_cd\_rcMin2 | 0.3054814 | -1.07406881 | -0.70677873 | 3 |
| arRayK25/scRayK25 | 0.2783823 | -1.09905008 | -0.69151514 | 3 |
| arRayK25/scRayK35 | 0.2804735 | -1.15233872 | -0.65209371 | 3 |
| arRayK25/scRay\_cat\_cd | 0.2926188 | -1.04946836 | -0.69302493 | 3 |
| arRayK25/scRay\_cat\_cd\_rcMin2 | 0.3143199 | -1.09993496 | -0.69381204 | 3 |
| arRayK25/scSoapK25 | 0.2533503 | -1.09178539 | -0.68885566 | 3 |
| arRayK25/scSoapK35 | 0.3021601 | -1.09815646 | -0.71451107 | 3 |
| arRayK25/scSoap\_cat\_cd | 0.1337285 | -0.66871210 | -0.84380966 | 3 |
| arRayK25/scSoap\_cat\_cd\_rcMin2 | 0.2765425 | -1.05246114 | -0.70886961 | 3 |
| arRayK35/ctOasesK25 | 0.5918760 | -1.33858981 | -0.37887843 | 3 |
| arRayK35/ctOasesK35 | 0.6009698 | -1.33483823 | -0.39018492 | 3 |
| arRayK35/ctOases\_cat\_cd | 0.5105021 | -1.22300723 | -0.43496905 | 3 |
| arRayK35/ctOases\_cat\_cd\_rcMin2 | 0.6074599 | -1.32553850 | -0.38628046 | 3 |
| arRayK35/ctRayK25 | 0.6349511 | -1.32365217 | -0.39918766 | 3 |
| arRayK35/ctRayK35 | 0.5618741 | -1.32617415 | -0.37125133 | 3 |
| arRayK35/ctRay\_cat\_cd | 0.6121202 | -1.27925636 | -0.39035064 | 3 |
| arRayK35/ctRay\_cat\_cd\_rcMin2 | 0.6495953 | -1.32599854 | -0.39041795 | 3 |
| arRayK35/ctSoapK25 | 0.5954398 | -1.31488834 | -0.37128324 | 3 |
| arRayK35/ctSoapK35 | 0.5513410 | -1.30044175 | -0.36826002 | 3 |
| arRayK35/ctSoap\_cat\_cd | 0.4980202 | -1.08887840 | -0.48208605 | 3 |
| arRayK35/ctSoap\_cat\_cd\_rcMin2 | 0.6246706 | -1.30912641 | -0.38698620 | 3 |
| arRayK35/scOasesK25 | 0.6247977 | -1.28485993 | -0.38909811 | 3 |
| arRayK35/scOasesK35 | 0.5947487 | -1.22500740 | -0.28670455 | 3 |
| arRayK35/scOases\_cat\_cd | 0.5809332 | -1.14356671 | -0.46032661 | 3 |
| arRayK35/scOases\_cat\_cd\_rcMin2 | 0.5902557 | -1.28136954 | -0.39230247 | 3 |
| arRayK35/scRayK25 | 0.6324846 | -1.32192509 | -0.38662448 | 3 |
| arRayK35/scRayK35 | 0.6042983 | -1.32548050 | -0.39096744 | 3 |
| arRayK35/scRay\_cat\_cd | 0.6193787 | -1.27173351 | -0.38379348 | 3 |
| arRayK35/scRay\_cat\_cd\_rcMin2 | 0.6380600 | -1.32124767 | -0.38432804 | 3 |
| arRayK35/scSoapK25 | 0.5653574 | -1.32483627 | -0.36956986 | 3 |
| arRayK35/scSoapK35 | 0.6006233 | -1.30115963 | -0.38745254 | 3 |
| arRayK35/scSoap\_cat\_cd | 0.4527097 | -1.06998008 | -0.47190941 | 3 |
| arRayK35/scSoap\_cat\_cd\_rcMin2 | 0.6079284 | -1.28281198 | -0.39175885 | 3 |
| ctEulK29 | 1.4722894 | -3.05070240 | 1.00317900 | 3 |
| aaMin2/ctALL/454Cap3 | 2.5486597 | 4.54488108 | -0.85673154 | 4 |
| aaMin2/ctOasesK25/454Cap3 | 2.8884683 | 1.75767648 | -1.29522476 | 4 |
| aaMin2/ctOasesK35/454Cap3 | 3.0083308 | 1.62430054 | -1.21968775 | 4 |
| aaMin2/ctOases\_cat\_cd/454Cap3 | 2.8758958 | 2.16751482 | -1.41075951 | 4 |
| aaMin2/ctOases\_cat\_cd\_rcMin2/454Cap3 | 2.8807531 | 2.23354530 | -1.36836450 | 4 |
| aaMin2/ctRayK25/454Cap3 | 2.5439872 | 3.27409361 | -0.78739373 | 4 |
| aaMin2/ctRayK35/454Cap3 | 2.9571681 | 1.98348853 | -0.67349946 | 4 |
| aaMin2/ctRay\_cat\_cd/454Cap3 | 2.6048213 | 3.44549032 | -0.67717328 | 4 |
| aaMin2/ctRay\_cat\_cd\_rcMin2/454Cap3 | 2.6010282 | 3.39846946 | -0.74380915 | 4 |
| aaMin2/ctSoapK25/454Cap3 | 2.5193938 | 4.02702175 | -1.32992011 | 4 |
| aaMin2/ctSoapK35/454Cap3 | 2.7571314 | 3.31120654 | -1.35347285 | 4 |
| aaMin2/ctSoap\_cat\_cd/454Cap3 | 2.6218729 | 4.27468360 | -1.35137214 | 4 |
| aaMin2/ctSoap\_cat\_cd\_rcMin2/454Cap3 | 2.6323456 | 4.23986162 | -1.46302283 | 4 |
| aaMin2/scALL/454Cap3 | 3.3566868 | 5.52902133 | -1.16983917 | 4 |
| aaMin2/scOasesK25/454Cap3 | 3.2234970 | 2.64649859 | -0.99521478 | 4 |
| aaMin2/scOasesK35/454Cap3 | 3.2363228 | 2.03748166 | -0.95781289 | 4 |
| aaMin2/scOases\_cat\_cd/454Cap3 | 3.3821720 | 3.29036926 | -1.11168129 | 4 |
| aaMin2/scOases\_cat\_cd\_rcMin2/454Cap3 | 3.3230395 | 3.43187605 | -0.96973784 | 4 |
| aaMin2/scRayK25/454Cap3 | 2.5664485 | 3.34791645 | -0.84001075 | 4 |
| aaMin2/scRayK35/454Cap3 | 2.9621313 | 2.07908051 | -0.64476739 | 4 |
| aaMin2/scRay\_cat\_cd/454Cap3 | 2.7006379 | 3.35922315 | -1.06024168 | 4 |
| aaMin2/scRay\_cat\_cd\_rcMin2/454Cap3 | 2.6907905 | 3.35576753 | -1.06877454 | 4 |
| aaMin2/scSoapK25/454Cap3 | 2.8328542 | 3.76680014 | -1.36518754 | 4 |
| aaMin2/scSoapK35/454Cap3 | 2.9037770 | 3.54319825 | -1.51233643 | 4 |
| aaMin2/scSoap\_cat\_cd/454Cap3 | 3.0024237 | 4.24453149 | -1.60776615 | 4 |
| aaMin2/scSoap\_cat\_cd\_rcMin2/454Cap3 | 2.9974926 | 4.20032234 | -1.71357875 | 4 |
| C.albicans | 9.5846266 | -0.35634371 | 1.90800135 | 5 |
| N.crassa | 6.5690503 | -4.01519677 | 3.06716150 | 5 |
| arMIRA/ctOasesK25 | 4.8107026 | -1.60004304 | 1.26033533 | 5 |
| arMIRA/ctOasesK35 | 4.8309434 | -1.59682739 | 1.22458179 | 5 |
| arMIRA/ctOases\_cat\_cd | 4.4479413 | -1.19165009 | 0.84468409 | 5 |
| arMIRA/ctOases\_cat\_cd\_rcMin2 | 4.8034856 | -1.62615098 | 1.23435777 | 5 |
| arMIRA/ctRayK25 | 4.9125561 | -1.61317191 | 1.32944753 | 5 |
| arMIRA/ctRayK35 | 4.9360693 | -1.65747106 | 1.28144528 | 5 |
| arMIRA/ctRay\_cat\_cd | 4.9036665 | -1.54230071 | 1.23287174 | 5 |
| arMIRA/ctRay\_cat\_cd\_rcMin2 | 4.8792573 | -1.67912518 | 1.28163037 | 5 |
| arMIRA/ctSoapK25 | 4.9900602 | -1.59625787 | 1.27104185 | 5 |
| arMIRA/ctSoapK35 | 4.9275109 | -1.57198991 | 1.21619109 | 5 |
| arMIRA/ctSoap\_cat\_cd | 4.3064405 | -0.84661873 | 0.61569536 | 5 |
| arMIRA/ctSoap\_cat\_cd\_rcMin2 | 4.9578407 | -1.60541065 | 1.24665296 | 5 |
| arMIRA/scOasesK25 | 4.9008362 | -1.37286792 | 1.49355250 | 5 |
| arMIRA/scOasesK35 | 4.8863290 | -1.40243902 | 1.49625508 | 5 |
| arMIRA/scOases\_cat\_cd | 4.5778647 | -0.68768512 | 1.36036500 | 5 |
| arMIRA/scOases\_cat\_cd\_rcMin2 | 4.9117252 | -1.60082044 | 1.36821376 | 5 |
| arMIRA/scRayK25 | 4.8867614 | -1.67048427 | 1.27738996 | 5 |
| arMIRA/scRayK35 | 4.9252320 | -1.68415975 | 1.28338146 | 5 |
| arMIRA/scRay\_cat\_cd | 4.8592430 | -1.58257666 | 1.23516081 | 5 |
| arMIRA/scRay\_cat\_cd\_rcMin2 | 4.9455152 | -1.57638271 | 1.34655496 | 5 |
| arMIRA/scSoapK25 | 4.8988912 | -1.65230583 | 1.34549001 | 5 |
| arMIRA/scSoapK35 | 4.9621260 | -1.56675408 | 1.24475485 | 5 |
| arMIRA/scSoap\_cat\_cd | 4.3636149 | -0.82967473 | 0.64583886 | 5 |
| arMIRA/scSoap\_cat\_cd\_rcMin2 | 5.0075487 | -1.58570986 | 1.27520755 | 5 |
| ctMIRA | 3.7819467 | -2.21554254 | 1.59280269 | 5 |
| ctMIRA\_ctEulK29\_rcCAP3 | 4.4736194 | -2.24029791 | 1.26032545 | 5 |

| PCA dimension 1 | | |
| --- | --- | --- |
| **Variables** | | |
| *Name* | *Correlation coef* | *p-valor* |
| DiffComplProts | 0.9666178 | 8.550270e-108 |
| Contigs500 | 0.9593754 | 2.653959e-100 |
| N50 | 0.9366102 | 1.879514e-83 |
| DiffProts | 0.9092316 | 4.872127e-70 |
| ComplOrtho | 0.8902303 | 4.966066e-63 |
| MeanContigLen | 0.8262800 | 1.712029e-46 |
| DuplOrtho | 0.8256612 | 2.284851e-46 |
| N90 | 0.7177403 | 6.004484e-30 |
| MeanContigCov | 0.6803979 | 5.975111e-26 |
| AllTransSize | 0.5474004 | 1.538235e-15 |
| Ns | 0.3339472 | 4.342911e-06 |
| MeanGapLen | -0.1948600 | 8.572259e-03 |
| MissAssembl | -0.2043579 | 5.789170e-03 |
| FragOrtho | -0.9031894 | 1.181237e-67 |
| **Factors** | | |
| *Name* | *R2* | *p-valor* |
| Program | 0.6981381 | 7.195731e-41 |
| Task | 0.3897774 | 9.785770e-15 |
| Technology | 0.2249552 | 8.267250e-10 |
| Kmer | 0.2271483 | 3.566675e-07 |
| **Categories** | | |
| *Name* | *Estimate* | *p-valor* |
| mira | 4.7640390 | 1.252631e-25 |
| rs\_454read/Ill | 1.9097259 | 2.424378e-15 |
| minimus | 1.7794195 | 5.580423e-05 |
| 29/25;35 | 0.9711299 | 5.872021e-05 |
| 29/35 | 1.0691816 | 8.707961e-03 |
| 29/25 | 0.8608128 | 1.484658e-02 |
| 454 | 2.2863545 | 4.555555e-02 |
| cat | -2.2942397 | 4.553239e-02 |
| SOAPdenovo | -2.8219667 | 4.546581e-02 |
| 35 | -2.5243166 | 3.485414e-02 |
| oases | -3.2541331 | 2.070879e-02 |
| cd-hit | -2.7954290 | 1.439406e-02 |
| merge | -2.8208319 | 1.232543e-02 |
| scaffolding | -3.1076125 | 5.763801e-03 |
| 25 | -2.8199001 | 1.718314e-03 |
| join | -2.5084918 | 1.266388e-03 |
| rs | -0.8725312 | 5.246865e-04 |
| 454-Ill | -0.4780410 | 1.930135e-06 |
| Ill | -3.8047227 | 2.337655e-10 |
| Ray | -1.2825483 | 3.281291e-13 |

| PCA dimension 2 | | |
| --- | --- | --- |
| **Variables** | | |
| *Name* | *Correlation coef* | *p-valor* |
| Contigs | 0.9124722 | 2.183004e-71 |
| AllTransSize | 0.8017348 | 7.201904e-42 |
| Ns | 0.7803849 | 2.403418e-38 |
| MissAssembl | 0.6489623 | 5.116425e-23 |
| DuplOrtho | 0.4119005 | 8.363370e-09 |
| DiffProts | 0.3678169 | 3.507767e-07 |
| MeanGapLen | 0.3270763 | 6.986188e-06 |
| Contigs500 | 0.2018253 | 6.438610e-03 |
| N50 | -0.2938301 | 5.952765e-05 |
| N90 | -0.4299766 | 1.533877e-09 |
| MeanContigLen | -0.4431286 | 4.187150e-10 |
| MeanContigCov | -0.7038684 | 2.169789e-28 |
| **Factors** | | |
| *Name* | *R2* | *p-valor* |
| Program | 0.8258495 | 3.377703e-61 |
| Kmer | 0.6100590 | 1.765515e-31 |
| Task | 0.4790774 | 2.632904e-20 |
| Technology | 0.3486670 | 2.113495e-16 |
| **Categories** | | |
| *Name* | *Estimate* | *p-valor* |
| minimus | 2.5827279 | 1.559402e-28 |
| 29/25;35 | 3.5825555 | 5.277061e-15 |
| Ill | 1.1434976 | 1.172046e-12 |
| join | 1.3138591 | 3.067497e-08 |
| cat | 3.1496886 | 4.142704e-06 |
| 29/25 | 3.0252549 | 6.466290e-05 |
| 454\_Ill | 3.9682443 | 2.632763e-04 |
| 25;35 | 0.9139553 | 1.828639e-03 |
| 29/35 | 2.3183798 | 2.163506e-03 |
| cd-hit | 1.8936995 | 3.267794e-03 |
| SC\_trs\_all | 3.8820534 | 5.061274e-03 |
| scaffolding | 0.4211019 | 9.303661e-03 |
| merge | 0.2839031 | 1.530104e-02 |
| rs\_all | 2.8979132 | 2.168254e-02 |
| 454 | -3.5708878 | 2.778899e-02 |
| 25 | -0.8442642 | 3.455830e-04 |
| mira | -1.9413098 | 3.422086e-05 |
| 35 | -1.1550997 | 2.027585e-07 |
| 454-Ill | -1.5408541 | 4.252707e-12 |
| rs\_454read/Ill | -2.9135165 | 7.789898e-14 |
| Ray | -1.4247604 | 1.380031e-16 |
